# The genomes of nucleocytoplasmic large DNA viruses: viral evolution writ large

**DOI:** 10.1099/mgen.0.000649

**Published:** 2021-09-20

**Authors:** Heli A. M. Mönttinen, Cedric Bicep, Tom A. Williams, Robert P. Hirt

**Affiliations:** ^1^​ Newcastle University Biosciences Institute, Faculty of Medical Sciences, Newcastle University, Newcastle upon Tyne, NE2 4HH, UK; ^2^​ School of Biological Sciences, University of Bristol, 24 Tyndall Ave., Bristol, BS8 1TH, UK; ^†^​ Present address: Institute of Biotechnology, Helsinki Institute of Life Sciences (HiLIFE), University of Helsinki, Viikki Biocenter 2, Helsinki 00014, Finland; ^‡^​ Present address: Université Clermont Auvergne, CNRS, LMGE, F-63000 Clermont Ferrand, France

**Keywords:** gene network, gene phylogenies, NCLDV, phylum* Nucleocytoviricota*, protein families

## Abstract

The nucleocytoplasmic large DNA viruses (NCLDVs) are a diverse group that currently contain the largest known virions and genomes, also called giant viruses. The first giant virus was isolated and described nearly 20 years ago. Their genome sizes were larger than for any other known virus at the time and it contained a number of genes that had not been previously described in any virus. The origin and evolution of these unusually complex viruses has been puzzling, and various mechanisms have been put forward to explain how some NCLDVs could have reached genome sizes and coding capacity overlapping with those of cellular microbes. Here we critically discuss the evidence and arguments on this topic. We have also updated and systematically reanalysed protein families of the NCLDVs to further study their origin and evolution. Our analyses further highlight the small number of widely shared genes and extreme genomic plasticity among NCLDVs that are shaped via combinations of gene duplications, deletions, lateral gene transfers and *de novo* creation of protein-coding genes. The dramatic expansions of the genome size and protein-coding gene capacity characteristic of some NCLDVs is now increasingly understood to be driven by environmental factors rather than reflecting relationships to an ancient common ancestor among a hypothetical cellular lineage. Thus, the evolution of NCLDVs is writ large viral, and their origin, like all other viral lineages, remains unknown.

## Data Summary

The authors confirm that all supporting data have been provided within the article through supplementary data files (Tables S1–S10, Figs S1–S11). The protein clusters, listed in Data S1, can be found at Figshare: https://doi.org/10.6084/m9.figshare.14884248.v1.

New protein families associated with NCLDVs curated in the Pfam database (release Pfam v33.1 and Pfam v34.0) can be found at: https://pfam.xfam.org/.

**Impact Statement**

Nucleocytoplasmic large DNA viruses (NCLDVs) currently include the most complex known viruses, characterized by the largest virions, genomes and corresponding protein-coding capacity, which overlap with the size and coding capacity of bacterial genomes. Here we systematically reviewed the published data on the evolution of NCLDVs. We have also expanded and systematically reanalysed protein clusters among NCLDVs to investigate the validity of previous phylogenies and hypothesized shared genes between NCLDVs and cellular life forms. Our reanalysis also defined 88 new Pfam NCLDV protein families. Notably our reanalysed protein clusters also create links between various data such as NCBI accession, Pfam identifiers and protein clusters identified by Yutin et al. in 2014. This review highlights in particular that NCLDVs share only a very small set of genes and that the tempo and mode of their evolution is very much reminiscent of viral, rather than cellular, genome evolution. We also review how the genome changes in different NCLDVs in response to environmental factors. This information will be of great interest to evolutionary biologists, virologists and molecular cell biologists, providing a comprehensive and critical view on the highly dynamic and intricate evolution of NCLDV genomes and genes.

## Introduction

The nucleocytoplasmic large DNA viruses (NCLDVs) are a group of dsDNA viruses that includes the largest known virions, genomes and number of annotated protein coding genes, which are sometimes called giant viruses with genome sizes that can exceed 2 Mbp and that were recently formally classified by the International Committee on Taxonomy of Viruses (ICTV) as members of the viral phylum *Nucleocytoviricota* [1] (https://talk.ictvonline.org/taxonomy) (Table 1). The very first giant virus was isolated from an air-conditioning system in 1992 during an outbreak of pneumonia in Bradford, UK [2]. This virus growing in the amoebozoan protist *Acanthamoeba polyphaga* was initially thought to be a rod-shaped bacterium, as it resembled a Gram-negative bacterium under the light microscope [2, 3] and it was subsequently named mimivirus referring to ‘mimicking microbe’. Mimivirus had several unique features that had not been previously observed among any virus. For instance, the virion size (750 nm) and the genome size (1182 kb) were larger than that of any known viruses at the time and the genome size and protein-coding capacity exceeded that of some bacteria. Particularly intriguing, the mimivirus genome encoded proteins that are broadly conserved among cellular genomes, including proteins related to the translation machinery [4]. These genes included the aminoacyl-tRNA synthetases for arginine, tyrosine and methionine, beta and beta′ subunits of DNA-dependent RNA polymerase, sliding clamp subunit of DNA-dependent DNA polymerase and 5′−3′ exonuclease [4]. Initial phylogenies of these genes suggested that the mimivirus homologues branched as the sister group to monophyletic eukaryotes, motivating the hypothesis that they might have evolved by genomic and organismal reduction from a lost fourth domain of cellular life [5]. However, subsequent analyses indicated that these initial results were probably phylogenetic artefacts, and that eukaryote-like genes on giant viral genomes were probably acquired independently from multiple origins from various eukaryotic hosts through independent lateral gene transfer (LGT) events, and therefore could not be justifiably integrated in a concatenated alignment to investigate a ‘universal phylogeny’ that includes the NCLDVs [6–8]. All these issues suggest that the NCLDV lineage does not represent a deep-branching fourth domain of life.

**Table 1.** The list of classified (according to ICTV classification) and unclassified NCLDVs considered in this review

Classified NCLDVs					
Virus class	Virus order	Virus family	Genome size (kb)	Number of predicted ORFs	Taxonomy of natural or experimental* or inferred** host
Megaviricetes	*Algavirales*	*Phycodnaviridae*	155–474	150–886	Alveolata, Chlorophyta, Haptophyta, Stramenopiles
	*Imitervirales*	*Mimiviridae*	617–1259	544–1120	Amoeba*, Stramenopiles
	*Pimascovirale*	*Ascoviridae*	119–186	119–180	Lepidoptera
		*Iridoviridae*	106–220	99–468	Arthropods, Fish, Amphibia
		*Marseilleviridae*	347–403	403–470	Amoeba*
Pokkesviricetes	*Asfuvirales*	*Asfarviridae*	170	152	Swines
	*Chitovirales*	*Poxviridae*	134–360	130–328	Arthropods, Vertebrates
**Unclassified NCLDVs**					
na	na	*Faustovirus*	460	451	Amoeba*
		*Kaumoebavirus*	351	465	Amoeba*
na	na	*Klosneuvirus*	1385–1570	1207–1545	Protist**
na	na	*Medusavirus*	381	461	Amoeba*
na	na	*Mollivirus*	652	523	Amoeba*
na	na	*Pithovirus*	610	467	Amoeba*
na	na	*Pandoravirus*	1909–2474	1487–2541	Amoeba*

The mimiviruses are now classified as a member of the family *Mimiviridae*, which belongs to the order *Imitervirales* and class *Megaviricetes* with all members of the phylum *Nucleocytoviricota* currently split among two classes*, Megaviricetes* and *Pokkesviricetes*, and a total of five orders and seven families. The other six families are named *Phycodnaviridae*, *Ascoviridae*, *Iridoviridae, Marseilleviridae, Asfarviridae* and *Poxviridae* [9] (Table 1). In addition, pandoravirus, pithovirus and mollivirus are also considered to be related to the NCLDVs, but they have not yet been formally taxonomically classified (Table 1) [8, 10–15] (https://talk.ictvonline.org/taxonomy/). The presently known NCLDVs infect a wide range of hosts including vertebrates, invertebrates, amoebae, dinoflagellates, rhizarians, Discoba, Stramenopiles, Chlorophyta and Haptophyta [3, 16–20]. In addition, recent metagenomic studies suggest that NCLDV-related viruses are ubiquitous in nature and are associated with most major eukaryotic lineages [21]. It should be noted that the natural host and host range for many NCLDVs are currently unknown. The lifecycle of NCLDVs is best described among poxviruses that replicate and assemble in the cytoplasm [22], and similarly mimiviruses seem to replicate exclusively in the cytoplasm [23]. In contrast, replication of asfarviruses and iridoviruses is initiated in the nucleus followed by a second stage in the cytoplasm, where virion assembly occurs [22, 24]. These characteristics explain the name of these viruses – the nucleocytoplasmic large DNA viruses (NCLDVs). The characteristics of NCLDV members are the presence of a dsDNA genome, which is typically over 100 kb, and they share five core genes comprising (i) major capsid protein (viral), (ii) D5 helicase (cellular and viral), (iii) DNA polymerase B (cellular and viral), (iv) A32-like packaging ATPase (viral) and (iv) viral late transcription factor 3 (also known as Poxvirus late transcription factor 3) [9, 25]. Although these five genes families were initially considered to represent core NCLDV gene families and support their monophyly, the D5-like helicase was shown to be replaced in phycodnaviruses by a gene derived from a bacteriophage(s), and multiple origins of DNA polymerase B in NCLDVs from various cellular donors cannot be excluded in some phylogenetic analyses [26]. Nonetheless, based on these shared features, the NCLDVs have been proposed to form a new viral order called the Megavirales [9] now formally classified as the phylum *Nucleocytoviricota* [1] (https://talk.ictvonline.org/taxonomy/).

Several studies on the evolution of NCLDV genomes have indicated that their genome size and protein-coding capacity were influenced by a combination of different mechanisms. Here we have revisited these issues through a pertinent combination of complementary approaches. Our data indicate that the protein content of NCLDVs is very specific to each virus family and the number of shared protein families between NCLDVs lineages is rather limited. Our results and published studies integrated here further highlight the plasticity and mosaic nature of NCLDV genomes, which are modified via a combination of gene duplications and deletions [27–30], lateral gene transfers [31, 32] and also probably *de novo* gene evolution [33].

## NCLDV comparative and evolutionary genomics – a view from protein families

### The classification of NCLDVs

Viruses are considered the most abundant and diverse biological entities on Earth [34], and viral metagenomics studies have indicated that the majority – perhaps 60–90 % [35] – of environmental viral sequences do not share significant sequence similarity with any presently known viruses [36]. Virus classification and taxonomy are challenging, because viruses do not universally share any homologous genes [37]. Thus, it is not possible to draw a universal tree of viruses based on core genes, and so the development of a single unifying classification is difficult. Instead, viruses are classified in various ways. For example, the Baltimore classification [38] divides viruses into seven classes based on the genome type and the method of mRNA synthesis: (I) double-strand DNA viruses (dsDNA), (II) single-strand DNA viruses, (III) double-strand RNA viruses, (IV) positive-sense single-strand RNA viruses, (V) negative-sense single-strand RNA viruses, (VI) single-strand RNA retroviruses and (VII) single-strand DNA retroviruses. NCLDVs form a small fraction of the known diversity of dsDNA viruses [39]. Taxonomically, viruses are divided into different taxonomic ranks by the ICTV [35, 40]. According to the ICTV criteria, viral species are described as a monophyletic group, the properties of whihc can be distinguished from those of other species [35]. Virus genera are defined as a group of virus species sharing a common character, a virus family is a group of genera that share a common character and a virus order is defined as a group of families sharing a common character [35]. The shared character can be, for example, genome type, mode of replication, virion morphology, host range, pathogenicity and sequence similarity [35, 41]. Due to the high variability of viruses, these criteria are applied in different ways for different viruses, even though sequence alignments and phylogenies are now one of the key factors considered in virus taxonomy [35]. In the case of NCLDVs, the current phylum *Nucleocytoviricota* (previously proposed order *Megavirales*) is based on the possession of a small universal and a larger ‘nearly universal’ protein-coding gene set [9]. We will investigate here the evidence supporting the shared evolution of the NCLDVs/*Nucleocytoviricota* and the different hypotheses for their origin(s) by considering both published and our expanded protein family-based analyses.

The universal protein-coding gene set of NCLDVs consists of five genes and the ‘nearly universal’ set comprises 50 genes that are found in most, but not all, NCLDVs. The genes were identified by applying a maximum-likelihood method [25, 26] in which gene gains and losses were mapped onto a reference phylogenetic tree based upon six concatenated genes (1 – DNA polymerase B, 2 – helicase II, 3 – packaging ATPase, 4 – D5 helicase, 5 – RNA polymerase A, 6 – RNA polymerase B) that are present on most of the NCLDV genomes and that share sufficient sequence similarity for phylogenetic analyses [11, 25]. The nearly 50 universal set of genes includes proteins needed for replication, transcription, DNA repair, recombination and nucleotide metabolism [25]. Notably, this set does not contain gene components for translation, which are only found among the largest members of the NCLDVs [4, 7, 42], but even these viruses do not encode either rRNA or ribosomal proteins. Furthermore, the largest NCLDVs can encode additional cellular genes, such as those encoding histones, glycolytic enzymes and enzymes of the citric acid cycle, further highlighting the complexity of these viruses [43–45]. The exact function of these different genes in NCLDVs is unknown and it could differ from that of the cellular genes, although histones in Melbournevirus were recently shown to be able to form nucleosomes analogous to those in eukaryotes [45]. Metabolism-related genes are relatively conserved in NCLDV lineages after acquisition from cellular genomes and they probably participate in reprogramming host cell metabolism to support virus reproduction [43]. The origins of NCLDVs has had two major competing hypotheses: the first one proposes that NCLDVs originated from the ancient fourth domain of cellular life through reductive evolution [4, 10] and the second suggests that most of the homologues of cellular life genes originated from the presently known domains of life through several independent LGT events [6, 7, 46].

### What is the evidence for the different hypotheses for NCLDV origins?

#### The hypothesis of a fourth domain of life

The hypothesis of NCLDVs representing the fourth domain of life was based on the phylogenetic analysis of the concatenation of informational genes from these viruses. Initial analyses used seven concatenated mimiviral genes identifying a separate branch from bacterial, archaeal and eukaryotic branches in the phylogenetic analyses, whereas most of the mimiviral genes did not have any detectable homologues in databases [4]. However, the rationale to concatenate these seven genes was not justified when detailed phylogenetic analyses supported distinct and multiple origins for these genes from various cellular lineages [6, 7, 46]. More generally, subsequent phylogenetic analysis of the NCLDV translation and transcription-related proteins supported the origin of individual genes from different domains of life and different lineages within these domains [7]. Furthermore, phylogenetic analyses of the recently identified klosneuvirus suggested acquisition of translational components from multiple origins from diverse eukaryotes [8]. Taken together, these different considerations indicate that there are no robust data supporting the origin of the NCLDVs through extreme reductive evolution from a fourth domain of cellular life.

#### The number of protein families shared among the NCLDVs is limited

The protein families among NCLDVs have been investigated in a few studies [7, 25, 47]. However, profile–profile-based searches have not been systematically used to investigate potential relationships between divergent NCLDV protein families, and some described protein families cannot be reproduced due to the limited information provided. In addition, the removal of GI numbers from the National Center for Biotechnology Information (NCBI) led to the loss of the link between protein families and the individual proteins making them up [11, 25]. Here, we have updated the protein dataset of NCLDVs and analysed it systematically by applying a combination of OrthoMCL [48] and profile–profile searches (Fig. S1, available in the online version of this article). Our dataset consisted of 99 complete NCLDV genomes, which had predicted ORFs in the NCBI database (Table S1). In total, our dataset covered 33 154 ORFs from ten NCLDV families or groups (Fig. S1). We identified 3464 protein clusters for NCLDVs, which contained 24 409 ORFs (Fig. S1). This number of protein clusters is lower than those from other studies (e.g. 5443 protein families in Yutin *et al*. [7]). Compared to work flow used by Yutin *et al*. [7], we used a more conservative approach by considering proteins from different clusters to belong to the same cluster, if the profiles of their respective initial distinct clusters overlapped over more than 50 % of their length with a probability above 95 % (Fig. S1), without any manual editing steps to generate a more rigorous and objective view on these protein clusters, which would have led to differences between our and Yutin *et al.*'s [7] clustering. Compared to Yutin *et al.* [7], we nearly doubled the number of new NCLDV genomes (49) that added 12 668 ORFs and that led to 963 new protein clusters, which were not identified in the Yutin *et al.* [7] study. Linkage to the Pfam families (v29.0) was initially identified for 932 (26.9 %) of the new clusters. In addition, based on our analysis, 88 new protein families were curated by the Pfam database (releases v33.1 and v34.0) [49] (Table S2). Notably, the file Data S1 (available from Figshare) contains NCBI accessions and their corresponding protein family identifiers including those identified by Yutin *et al.* [7].

**Fig. 1.**
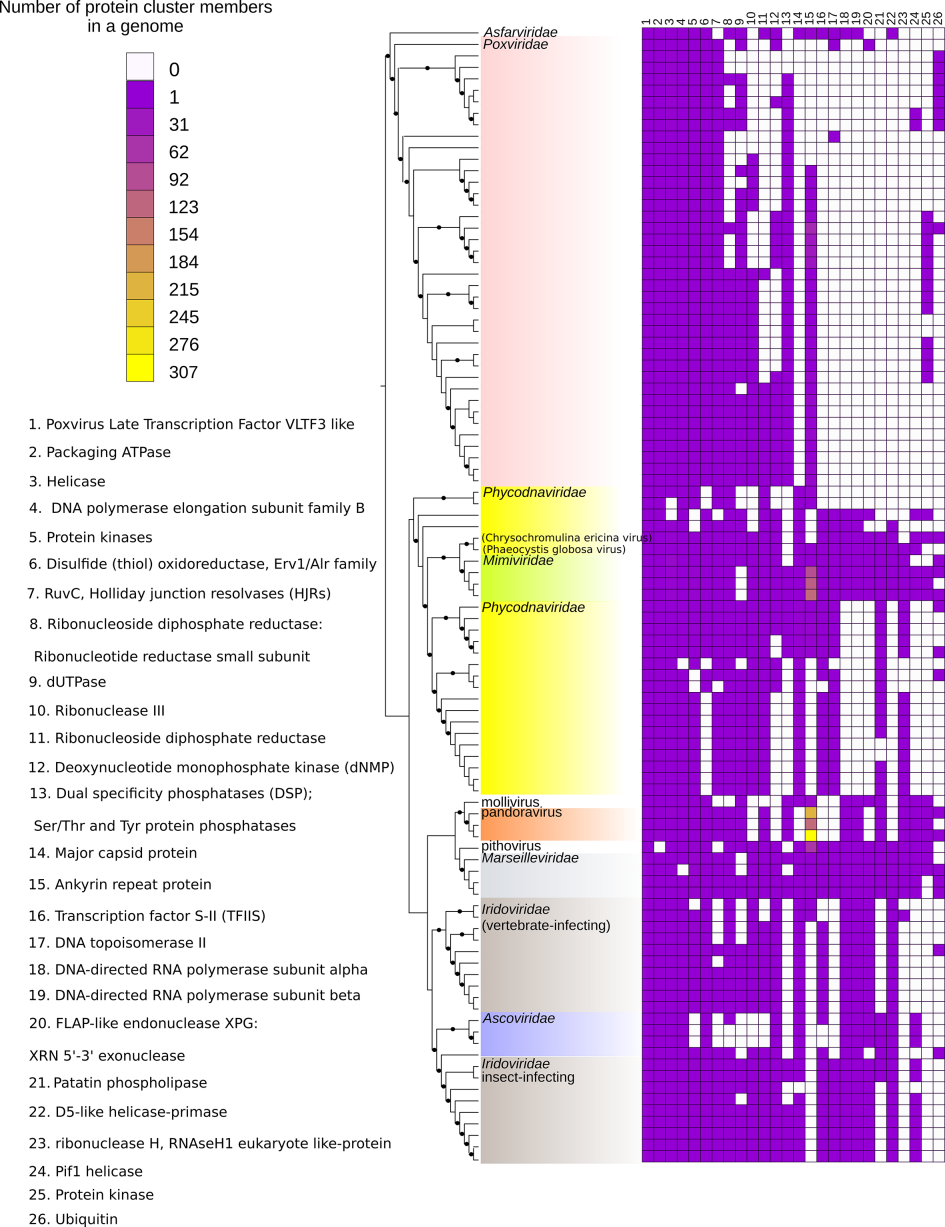
The presence–absence tree of NCLDVs and distribution of the 26 most shared protein clusters in NCLDVs. The tree is based on the presence–absence of the 3464 protein clusters. The protein clusters were made as follows. The tree was reconstructed from binary data with GTR2 model [95] with an ascertainment bias correction model [96] and 1000 ultrafast bootstraps [97] using IQ-tree [98]. Branches are marked with a black dot if the branch is supported at >95 %. The presence and number of protein cluster members are shown in a heatmap for the 26 most shared protein clusters that are present in more than six virus families or groups.

The majority of the protein families (2256, 65.1 %) were present in one to three genomes, with only 7.9 % of protein families found in more than ten genomes (Fig. S2). Only one protein cluster was shared by all the analysed NCLDV genomes, suggesting that most of the protein families are specific for a single virus or a group of closely related lineages (Family 1 in Fig. 1, viral late transcription factor 3). Despite the use of profile–profile-based searches, we cannot exclude the possibility that a number of related protein families were missed due to excessively low sequence similarity among some of the most divergent viral genes. Similarly, some excessively divergent viral proteins could not have been identified as members of any of the identified families. The ORFs of more recent NCLDVs and NCLDV-like genomes that were published after our new protein clusters were generated were blasted against the protein cluster sequences and homologous ORFs identified and these results are summarized in Table S3.

To evaluate the potential phylogenetic signal in gene content among NCLDVs, we inferred a phylogeny from protein cluster presence–absence data (Fig. 1, see legend for details). The resulting tree resolved the majority of established viral families with high bootstrap support, except the families *Phycodnaviridae* and *Iridoviridae* (Fig. 1). From the analysed data set, 26 protein clusters were shared by more than six virus family/groups, forming a ‘most commonly shared gene set’ for NCLDVs (Fig. 1, Table S4). Phylogenetic trees were generated for each of the five most widely distributed protein clusters among NCLDVs and respective homologues from cellular and other viral origins (Figs S3–S7, Table S5) to evaluate the origins of these genes.

Only the viral late transcription factor 3 was shared by all the virus genomes analysed (Fig. 1). In contrast to previous studies, DNA polymerase B elongation subunit was not found in any of the NCLDVs, which is due to the marseillevirus DNA polymerase no longer being in the NCBI protein database (from 18 July 2016). DNA polymerase B is apparently missing also from the genome of Yellowstone phycodnavirus 3 (a metagenomic assembly). The other protein families that are found in some but not all NCLDVs are poxvirus protein A23 (packaging ATPase) disulphide oxidoreductase, Erv1/Alr family and poxvirus protein A22 (RuvC, Holliday junction resolvases) (Fig. 1). We excluded helicases and protein kinase families from our phylogenetic analyses as these are complex protein families characterized by diverse, and often complex, domain compositions and diverse configurations. Despite the fact that a major capsid protein is one of the core genes among NCLDVs, it was not identified widely among NCLDVs in this study due to the limited sequence similarity of major capsid protein homologues between the family *Poxviridae* and other NCLDVs [50]. Notably, previous evidence for the homology between the major capsid proteins among NCLDVs was based on their structural similarity [51]. The presence of a major capsid protein-like sequence in pandoraviruses is still being debated with currently no experimental evidence for such proteins [33, 52].

#### The most commonly shared protein clusters in NCLDVs do not have monophyletic origins with the domains of cellular life

The phylogenies for the five most conserved protein families suggest that viral late transcription factor 3, packaging ATPase and Holliday junction resolvase families are probably monophyletic in NCLDVs, as the phylogenies of these genes do not contain a monophyletic group of cellular or other virus genes (Figs S3–S7). Few eukaryotic homologues involved in these phylogenies are from different taxonomic clades and are scattered across different parts of the tree, and thus they have probably acquired genes from a virus via LGT into eukaryotic genomes; for example, the *Ectocarpus siliculosus* genome is known to contain an inserted virus in its genome and the *Ectocarpus siliculosus* ORFs in these trees originate from this insertion [53] (Figs S3, S4 and S7). Virus-to-eukaryote LGTs are likely to be notably more common than previously thought and recently widespread NCLDV insertions are described in genomes of diverse green algae, some forming up to 10 % of all ORF content of a given algae genome [54]. This suggests that these viruses can represent an important source of new genetic material for some of their hosts. More generally, this adds the NCLDVs to the growing list of viruses that can contribute to new genetic material to their hosts through virus-to-host LGTs [55].

In previous studies, viral late transcription factor 3 and packaging ATPase have been considered monophyletic due to a lack of close homologues outside of the NCLDVs [26]. However, recently identified yaraviruses, which are either highly reduced and divergent NCLDVs or, more probably, the first non-NCLDV isolated from *Acanthamoeba* species, has also an ATPase most similar to the mimivirus homologue [56]. A phylogeny of the yaravirus major capsid protein [56] is not compatible with that of the ATPase phylogeny, suggesting that an LGT is the most likely origin for the yaravirus ATPase. Notably, our study is the first that shows a phylogeny for NCLDV Holliday junction protein covering at least one protein from each virus family and group (except the family *Asfarviridae* formed of only one species and unclassified molliviruses and pithoviruses), suggesting that among NCLDVs the Holliday junction proteins are also probably monophyletic (Fig. S4).

The eukaryotes in the disulphide (thiol) oxidoreductase-based phylogeny appear monophyletic and the acquisition of disulphide (thiol) oxidoreductase occured early in the evolution of NCLDVs. The oxidoreductase-based phylogeny is the only one in which the gene is widely shared with cellular life and their monophyly in NCLDVs is strongly supported (Fig. S7). In contrast, the DNA polymerases are not monophyletic, as those of the families *Poxviridae* and *Asfarviridae* were located in separate branches. Interestingly, the rest of the NCLDV polymerases and polymerases of the family *Herpesviridae* (see Table 2) are located in the same highly supported branch with eukaryotic DNA polymerases δ and ζ (Fig. S5). This suggests that the origin of the NCLDV homologues may lay among eukaryotic polymerases, especially polymerase δ that has been suggested to be responsible for leading- and lagging-strand synthesis in eukaryotes [57]. Also in previous studies, NCLDVs (except *Poxviridae* and *Asfaviridae*) were most similar to eukaryotic DNA polymerases δ and ζ, although this connection was not well supported [26]. The strongest evidence for the evolutionary relationship between NCLDVs and eukaryotic δ polymerases is provided from recently the identified medusavirus, which forms a well-supported sister group to the eukaryotic polymerase δ in a Bayesian phylogenetic analysis [44].

**Table 2.** Other virus families considered in the review and their links with NCLDVs

Virus kingdom	Virus phylum	Virus class	Virus order	Virus family (or virus-related element)	Description	Link to the NCLDV
na	na	na	na	*Baculoviridae*	A dsDNA virus family infecting insects	Shares several similar genes especially with insect-infecting NCLDVs. A DNA polymerase is similar to NCLDVs.
*Heunggongvirae*	*Peploviricota*	*Herviviricetes*	*Herpesvirales*	*Herpesviridae*	A dsDNA virus family infecting vertebrates.	A DNA polymerase similar to NCLDVs
na	na	na	na	Politons	Large and complex transposable elements, which are found among eukaryotes. They have a conserved set of genes: protein-primed type B DNA polymerase (pPolB), retroviral family integrase, A32 -like ATPase, adenovirus-type cysteine protease and two capsid proteins.	A suggested origin for NCLDVs and virophages. The packaging ATPase 32-like protein and the major capsid protein have a common origin with phycodnavirus. The minor capsid protein is similar to *Mimiviridae* and *Phycodnaviridae*.
*Bamfordvirae*	*Preplasmiviricota*	*Maveriviricetes*	*Priklausovirales*	*Lavidaviridae* (currently classified virophages)	Small dsDNA virus that needs co-infection of a giant virus. The replication of virophage is dependent on the giant virus replication system. Most of the virophages have a small subset of conserved genes: Minor and major capsid proteins, A32-like ATPase, cysteine protease, primase-superfamily three helicase and zinc-ribbon domain protein.	A suggested origin from Politons. Distant structural and sequence similarity to Polintons and a NCLDV jelly-roll capsid. A minor capsid protein is similar to NCLDVs, Polintons and tectiviruses.
*Bamfordvirae*	*Preplasmiviricota*	*Tectiliviricetes*	*Kalamavirales*	*Tectiviridae*	dsDNA bacteriophage	A suggested origin for Polintons. The DNA polymerase is similar to polintons. Structural similarity between the major capsid protein and NCLDV jelly-roll capsid. Protein sharing distant sequence similarities with minor capsid proteins of mimiviruses and phycodnaviruses
na	na	na	na	Yaravirus	Recently described amoeba-infecting virus. Either highly derived NCLDV or the first non-NCLDV *Acanthamoeba* spp. infecting virus.	The yaravirus has a major capsid protein and ATPase similar to NCLDVs.

#### In total, 55 % of the NCLDV proteome is formed of species-specific and virus-family-specific genes

**Fig. 2.**
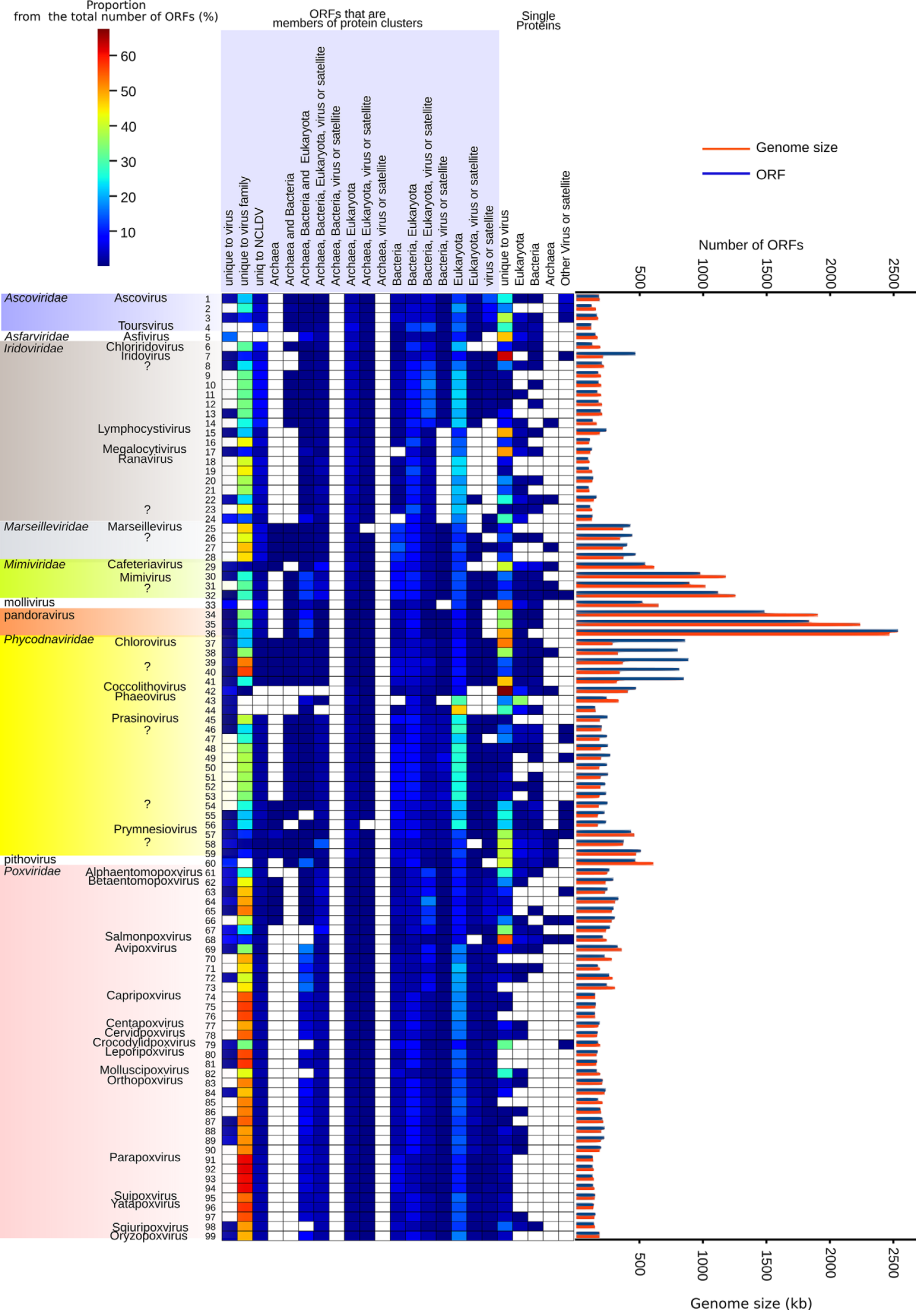
The ORF content in NCLDVs and percentage of homologues outside NCLDVs. The ORF content of each NCLDV is mapped on the heatmap, reflecting the percentage of ORFs belonging to a certain category listed on the top. The ORFs are classified into two groups if they are members of (a) a protein cluster or (b) single proteins that are not members of protein clusters. Both of these categories are divided into smaller subcategories, if (1) protein clusters (or single protein) are found only within the NCLDV or (2) if homologues are found outside NCLDVs (based on the best blast hits). Every ORF can belong only to one classification. The presence of a protein cluster in cells and other viruses is based on the best protein blast hits (blast version 2.2.31+) of each protein cluster member outside NCLDVs. The NCLDV protein cluster members were blasted against the NCBI non-redundant protein database, which was downloaded on 18 July 2016. The used e-value cut-off for protein blast hits was 10^−5^. Colours reflect the proportion of ORFs in the NCLDV genome falling into a classification. On the right the NCLDV genome size (kb) and number of ORFs are given as a histogram. The numbers on the left of the heatmap refer to the corresponding NCLDV genome in Table S1.

**Fig. 3.**
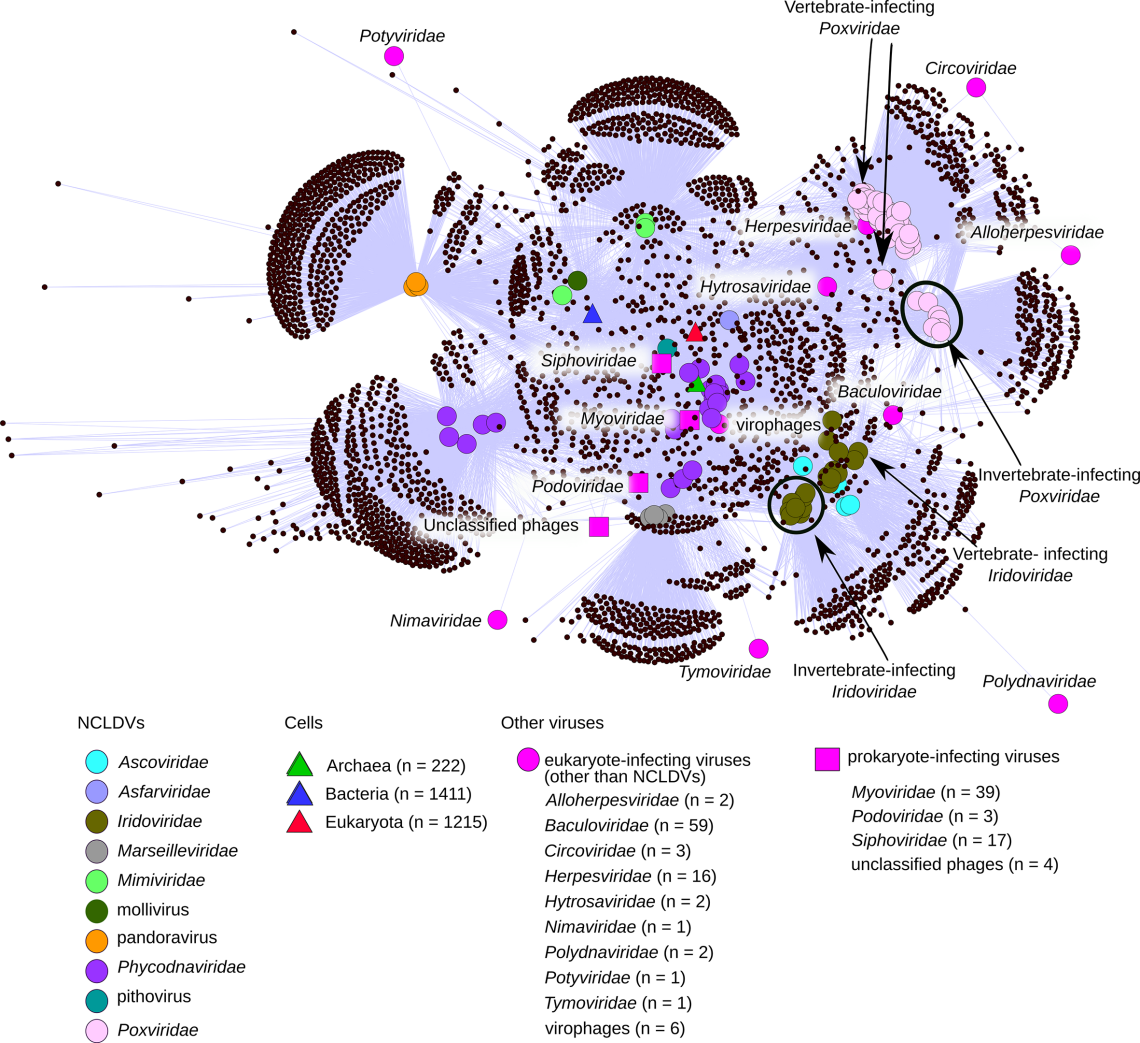
Bipartite network of shared protein clusters. The bipartite network is drawn for NCLDV protein clusters that shared by at least by two NCLDV genomes or NCLDV and other virus family or a cell. Protein clusters are depicted with small black circles that are connected to NCLDV genomes or cell proteins or other viruses (large nodes). The ORFs included in a cell or other virus nodes are indicated in parenthese. An NCLDV genome is linked to a protein cluster, if the genome contains at least one member of the particular protein cluster. The presence of a protein cluster in cells and other viruses is based on the best protein blast hits (version 2.2.31+) of each protein cluster member outside NCLDVs. The NCLDV protein cluster members were blasted against the NCBI non-redundant protein database excluding NCLDV members, which was downloaded on 18 July 2016. The used e-value cut-off for protein blast hits was 10^−5^. The bipartite network is visualized using an organic layout in Cytoscape v2.8.0 [99].

Most of the ORFs in NCLDV genomes are either unique, species-specific genes or form unique protein clusters within the NCLDV virus family (55 % of all the annotated proteins) (Figs 2 and 3). In addition, 323 (9.3 % of all clusters) protein clusters (derived from 1700 ORFs – 5.1 % of all ORFs) are shared only within NCLDV virus families (two or more). They do not show similarity towards proteins from any other viruses or cellular lineages. Some of these proteins may have homologues among cellular life or other viruses but the sequence similarity is too low to establish that relationship from primary sequence comparisons. The function is unknown for 90.4 % of the NCLDV-specific protein clusters. Only 9.6 % of these have Pfam annotation, and many of these annotations are from protein families specific for the *Poxviridae* (Table S6), which is the best-studied virus family among NCLDVs. The annotated functions for the ten most numerous NCLDV-specific proteins (all annotated for the family *Poxviridae*) are related to cell entry, transcription and inhibition of apoptosis (Table S6a). The high number of virus entry proteins is in line with previous observations that proteins needed for the interaction with the host are less conserved and more lineage-specific compared to the other functional categories [58]. The proportion of unique protein clusters in an NCLDV virus genome from all the ORFs does not correlate with genome size or ORF number (Figs 2 and S8), even though LGT or gene duplications have been emphasized to affect the expansion of genome size in the case of the largest NCLDVs [59].

The homologous ORFs in the more recently identified NCLDV genomes indicate at least 20 % of the ORFs have homologues in other NCLDVs (Table S3). In contrast, Yaravirus, probably the first non-NCLDVs virus identified in *Acanthamoeba* species, share with NCLDVs only five proteins (Table S3), which are the previously identified ATPase, the major capsid protein, a Yqaj-like viral recombinase protein and two hypothetical proteins related to one protein family from *Marseilleviridae* [56].

#### Protist-infecting NCLDVs possess the highest number of shared protein families with eukaryotes and bacteria

At least one member of 1032 protein clusters out of 3464 gave a significant protein blast hit outside NCLDVs. From this set 698 gave a blast hit to eukaryotic proteins outside NCLDVs, and 439 to bacterial and 51 archaeal proteins (Figs 2 and 3). To estimate how similar the shared protein cluster sets are between the two NCLDV genomes, or one genome of one NCLDV and other taxa (cellular life domain or another virus family), we built a network and calculated a Jaccard index that measures the degree of shared protein clusters between two NCLDV genomes or between one NCLDV genome and other taxa. The NCLDV family that has the most similar protein content toward eukaryotes are members of *Mimiviridae*, whereas animal-infecting viruses share notably fewer with the exception of the insect-infecting *Iridoviridae* (Fig. S9). Mimiviruses also share the most similar protein cluster set to bacteria. Also, other amoeba-infecting viruses and algae-infecting viruses, including pithovirus, family *Marseilleviridae*, mollivirus, pandoravirus and family *Phycodnaviridae* members share similarities in protein content to *Bacteria*, whereas protein families of animal-infecting share only little similarities to *Bacteria*. However, if the ORF contents are compared instead of protein clusters, a subset of phycodnaviruses appears to carry he highest percentage of similar ORFs to eukaryotes (Fig. 2). Phaeoviruses, such as *Ectocarpus siliculosus* virus, insert into the host genome as a part of their life cycle, which explains the high percentage of similar genes to eukaryotes [53]. In addition, recent studies show that several green algae genomes contain NCLDV-origin segments, suggesting that gene flux from virus to eukaryotic host is not limited to *Ectocarpus siliculosus* [54]. The gene flux between virus and host has also affected phycodnavirus genomes. For example, prasinovirus genome ORF content has apparently been affected more substantially by LGT than that of mimivirus [28], and the identified LGTs include unique gene transfers in prasinoviruses that have been acquired from the host prasinophyte [60]. Notably, the proportion of ORFs similar to cellular life does not correlate with genome size or number of ORFs (Fig. 2).

The phagocytotic lifestyle of the host cell is proposed to form a melting pot of genes, which could explain the high number of bacterial genes in amoeba-infecting NCLDVs [59]. However, the proportion of bacterial ORFs in pandoraviruses and marseilleviruses are similar to algae-infecting viruses, whose host range also covers non-phagocytosing species (Figs 2 and S9). For example, the *Chrysochromulina* are phagocytotic [61], but *Ostreococcus* species are autotrophic [62]. Thus, phagocytosis may not be the only explanation for the presence of bacterial genes in NCLDVs.

#### LGT between NCLDVs and Archaea

Jaccard indices of protein sets of a few *Ascoviridae*, *Asfarviridae* and *Phycodnaviridae* members indicate similarity to archaeal genes (Fig. S9). We investigated these families in detail as genes of archaeal origin were not described in NCLDVs. Most of the shared protein clusters appear to originate from LGTs between NCLDVs and bacteria or eukaryotes rather than between NCLDVs and Archaea. However, one protein cluster in chlorellaviruses (cluster_852 in Data S1) received a statistically significant protein blast hit [against the non-redundant (NR) database] from a non-histone chromosomal MC1 family protein of Euryarchaeota (* Methanocella conradii *, NCBI proteinid: WP_014405649, evalue 3e^−6^, date: 21f February 2021) that is needed for thermal stability of DNA in Archaea [63]. Also, the other hits outside NCLDVs are from Euryarchaeota, even though they are not statistically significant. To our knowledge, this is the first finding suggesting a candidate LGT from Archaea to NCLDVs.

#### Host type affects the protein content in NCLDVs

The protein content of insect-infecting NCLDV family members differs from those of vertebrate-infecting viruses (Figs 3, S9 and S10), which was unnoticed in previous network-based analyses probably due to low sampling of insect-infecting virus family members [43]. Two NCLDV families, *Poxviridae* and *Iridoviridae*, infect both vertebrates and inverterbrates. In the bipartite network analysis, *Poxviridae* and *Iridoviridae* cluster into two groups, in which one contains insect-infecting family members and the other vertebrate-infecting ones. Also, the Jaccard index profiles (Fig. S9) differ between insect- and vertebrate-infecting members of *Iridoviridae* and *Poxviridae*, indicating distinct protein content between these viruses. In addition, insect-infecting NCLDVs share unique ORFs with non-NCLDV dsDNA viruses of the insect-infecting *Baculoviridae*, *Polydnaviridae* and *Hytrosaviridae* families (Table S7, Fig. S10). Most of the 23 protein clusters that are uniquely shared within insect-infecting NCLDVs and non-NCLDV viruses have an unknown function (14 protein clusters in total). However, some of the annotated ORFs are known to manipulate the host, such as apoptosis inhibition (Table S7). Gene sharing between the insect-infecting *Poxviridae* and *Baculoviridae* has been observed before [64]. It is likely to be a consequence of LGTs directly between co-infecting viruses or that diverse viruses infecting the same host tend to acquire similar genes directly from the host or other pathogens inside the host [65]. For example, fusolin is needed for peroral infection in insects and it is shared by insect-infecting pathogens covering poxviruses, baculoviruses, bacteria and amoeba [65]. Thus, distantly related virus families may evolve towards similar genomic adaptation in the shared environment, is this case insect hosts. More frequent gene exchange between insect-infecting viruses is also supported by the 20 single NCLDV ORFs that have homologues in other insect-infecting viruses, especially those of *Baculoviridae* (Tables 2 and S7). Interestingly, only five protein clusters and two singleton ORFs were shared between vertebrate-infecting NCLDVs and other vertebrate-infecting viruses (Table S8), which is surprising as these represent one of the best sampled genomes among NCLDVs. The only vertebrate-infecting NCLDV families for which we could observe evidence for gene-sharing infected fish or amphibians: *Iridoviridae* (Ranavirus genus and scale drop disease virus) and *Poxviridae* (Salmon gill virus), which are members of different classes, respectively Megaviricetes and Pokkesviricetes.

#### Viral gigantism is polyphyletic

The phylogeny based on the presence–absence of the protein clusters suggests that large genomes have evolved at least twice among NCLDVs. The family *Mimiviridae* and pandoraviruses have the only members among NCLDVs with a genome size greater than 1 MB. Members of *Mimiviridae* are located within smaller members of *Phycodnaviridae* as a sister group to *Chrysochromulina ericina* virus and *Phaeocystis globosa* virus with a strong bootstrap value, suggesting a common ancestor (Fig. 1). In the five genes, the most broadly distributed among NCLDVs, pandoraviruses and mimivirus did not cluster together in any of the phylogenies. Instead, the *Mimiviridae* proteins were grouped with *Chrysochromulina ericina* virus and *Phaeocystis globosa* virus in a highly supported branch in trees based on packaging ATPase and disulphide (thiol) oxdidoreductase, supporting that the similarities in protein content of *Mimiviridae* and *Phycodnaviridae* also have a common evolutionary history (Figs S3–S7). Our observations are consistent with previous results indicating that the closest relatives of *Mimiviridae* are *Chrysochromulina ericina* virus and *Phaeocystis globose* virus [7, 66], implying that giantness in pandoraviruses has evolved independently [7]. Previously, pandoraviruses have been suggested to be highly derived phycodnaviruses based on the phylogenies of six (1 – DNA polymerase B, 2 – D5 primase-helicase, 3 – viral late transcription factor 3, 4 – A32 packaging ATP, 5 – DNA-directed RNA polymerase, subunit α and 6 – DNA-directed RNA polymerase, subunit β) broadly distributed NCLDV genes (from a total of 1487–2541 ORFs depending on the pandoravirus species) in which pandoraviruses grouped with phycodnaviruses [11]. Our gene trees supported the relationship of pandoravirus to phycodnaviruses in two trees, in which the closest phycodnaviruses are *Emiliana huxleyi* virus (packaging ATPase, Fig. S4) and prasinoviruses (Holliday junction resolvase, Fig. S7). In the other trees, the pandoraviruses did not group with any other virus family or groups with strong support values, except in the phylogenies for DNA polymerase B (Fig. S7) and disulphide oxidoreductase (Fig. S6) in which they clustered with mollivirus, as they did in the protein cluster-based phylogeny (Fig. 1). The Jaccard index profile of the pandoraviruses also indicates that they are most similar to mollivirus (Fig. 1 and S10), and both of these viral lineages are part of a larger network and do not form a separate subnetwork as described previously [43]. In contrast, the mimiviruses, the other lineage containing the largest NCLDVs genomes, cluster within the phycodnaviruses in the protein cluster-based phylogeny (Fig. 1); four individual gene phylogenies are also consistent with this relationship (Figs S4–S7). These data further support independent genome gigantism [7] for both the mimiviruses and pandoraviruses.

### NCLDV relationship to polintons, virophages and bacteriophages

NCLDVs have been suggested to have originated from the large transposable elements in the eukaryotic genome called polintons that in turn may have originated from inserted bacteriophages in early eukaryotes [67]. This hypothesis was put forward based on gene similarity network analyses in which polintons and some bacteriophages shared similarities between five core genes [39, 67]. There is also a competing scenario that polintons may have originated from inserted virophages [68, 69], which are small infecting viral-like particles of the largest NCLDVs [67]. The first hypothesis is supported by the observable similarities in protein sets on the sequence or structural level [39, 67]. The latter hypothesis [68] relates to the biology and function of the virophage and the similar gene order between the virophage and polinton (Table 2).

Polintons are 15–20 kb in length and they have been observed at least in Chroalveolata, Excavata, Rhizaria and Uniconta [70]. They also have a small set of conserved or nearly conserved genes such as protein-primed type B DNA polymerase (pPolB), retroviral-like (RVE) family integrase, FtsK-like ATPase, adenovirus-type cysteine protease and two putative capsid proteins [39, 67, 71]. The conserved proteins in polintons are similar to those of virophages. Virophages have a dsDNA genome of 18 kb-30 kb with 20–34 ORFs [72]. Most of the virophages share a conserved set of genes consisting of major capsid protein, minor capsid protein, FtsK-HerA family DNA-packaging ATPase, cysteine protease, the primase-superfamily 3 helicase and a zinc-ribbon domain protein [72]. Based on this gene set, virophages have been suggested to form a monophyletic group that justifies the classification of virophages into one newly recognized virus family [72].

In our analysis, 42 protein clusters were shared by at least one NCLDV and one bacteriophage (Table S9) and eight protein clusters were shared by at least one NCLDV and one virophage (Table S9). These two sets of protein clusters did not overlap. Thus, our analysis supports the sporadic relationship of NCLDVs and bacteriophages, as well as NCLDVs and virophages. Two of the protein clusters shared with bacteriophages are included in the 26 ‘nearly universal’ protein set (Table S9) and none of those are connected with virophages (Table S10) (Fig. 3). In addition, seven singleton ORFs in NCLDVs received the best blast hit from a bacteriophage, suggesting more recent gene sharing between NCLDVs and phages.

The presence of the capsid protein ORFs in polintons has led to the idea that polintons might actually be viruses and they could be the origin for both NCLDVs and virophages [67]. The first hypothesis proposes that NCLDVs developed from polintons when they gained a capping apparatus and DNA-dependent RNA polymerase from their host and adapted to a lifestyle, at least partly, outside the nucleus [67]. Later, NCLDVs gained helicase-primase, and the family B protein-primed DNA-dependent DNA polymerase was replaced by family B nucleic-acid-priming DNA-dependent DNA polymerase. The evidence for this hypothesis is based on a set of sequence and structural comparisons for the shared proteins between NCLDVs, polintons, virophages, bacteriophages, bidnaviruses (ssDNA virus) and adenoviruses (dsDNA virus) [67]. Phylogenetic analysis suggests the monophyly for the protein-priming family B DNA polymerases of polintons, adenoviruses, bidnavirus, cytoplasmic plasmids and mitochondrial plasmids, and that they all originated from phages [67]. The A32-like packaging ATPases of NCLDVs and polintons belong to the same A32 protein family, which belongs to the same A32 clade with the phage tectivirus PRD1 packaging ATPases P9 [73]. However, phylogenetic analysis of the ATPases does not support the common origin for NCLDV ATPases from polintons [74]. The putative major capsid in polintons shares a common origin with the capsid of phycodnavirus and PgVV virophage [70]. Structural comparisons and other sequence comparison methods indicate that NCLDV jelly-roll capsid proteins are most similar to that of Sputnik virophage, consistent with the dependence of virophages on NCLDVs, whereas the phage tectivirus PRD1 and human adenovirus are more distantly related to these [52, 75]. A minor capsid protein similar to tectivirus phage has been predicted from polintons, mimiviruses and phycodnaviruses by PSI-blast analysis [67]. However, these connections were not found in our analyses due to our more conservative sequence analysis approach.

The combination of shared gene order and primary sequence similarities between the mavirus virophage and a politon from the slime mould *Polysphondylium pallidum* is consistent with the latter hypothesis [68, 76]. In addition, some virophages have been demonstrated to insert into the genome of eukaryotes infected by NCLDVs and to control the transcription levels of the NCLDVs, and by doing so improve the survival of the eukaryotic host from giant virus infections at the population level and hence have the potential to evolve into transposable elements [68, 69].

### The giantness of NCLDVs – why become bigger?

As discussed earlier, genome giantess in NCLDVs developed twice, this assuming that the pandoraviruses are NCLDVs [10] as supported by our protein cluster analysis (Fig. 1). Genome and virion sizes are very variable within NCLDVs, ranging from 100 kb to 2.4 Mb. The largest NCLDVs, so-called giant viruses, have genomes larger than 500 kb (mimivirus, pandoravirus, pithovirus and mollivirus). Most tested giant viruses were shown to be capable of infecting amoeba, but their natural host or host range is often unknown. NCLDVs with the smallest genomes are those infecting vertebrates and insects (*Ascoviridae*, *Iridoviridae* and *Poxviridae*). Thus, one of the most fascinating questions regarding NCLDV genomes is why some of those have become so large; for instance, are they only infecting amoeba?

#### The size of NCLDV genomes can change quickly as a response to environmental changes

The large genomes of certain NCLDVs have been linked to the presence of bacteria in their amobea host [59]. Interestingly, the *Acanthamoeba* mimivirus genome reduces when *Acanthamoeba castellani* is grown in axenic culture without intracellular bacteria [59]. However, the reason behind this is unclear. The mimivirus genome shrunk by 16% when it was subcultured 150 times in bacteria-free amoeba cultures [59]. This shrinkage affected the tips of the genomes in particular, in which duplicate genes are frequently located. The largest deletions covered 155 ORFs and in addition 205 ORFs experienced internal deletions. These modifications led to the loss of mimivirus fibres on the virion surface that are made of glycoproteins, and the loss or mutation of 63 ankyrin-domain-containing proteins, seven serine/threonine kinases, 16 proteins involved in DNA replication, repair or recombination, and nine proteins involved in carbohydrate metabolic function and arginyl-tRNA synthetase [59]. These gene losses of the mimivirus genome were potentially explained by a loss of competition inside the amoebal cell between mimivirus and bacteria [59]. Consistent with this hypothesis, it has been previously observed that the physiology of the amoeba is different when grown either axenically or with endobacteria [77, 78]. For instance, it has been reported that in the absence of bacteria *Acanthamoeba* species were characterized by a reduction in virulence and secretion of proteases, the loss of encystation capacity and changes in drug susceptibility [77, 78]. Relatively larger genome sizes may not only be limited to amoeba-infecting viruses, as this was also observed among endobacteria for amoeba, which have been estimated to have 15–43% larger genomes compared to intracellular human bacterial pathogens [79].

#### The genome size of NCLDVs can change due to insertions, deletions and duplications

The variance in NCLDV genome size and genome content is strongly affected by duplication and LGT events [28]. In RNA viruses, the major source of genome variation and adaptation is the high mutation rate [80]. However, the mutation rate in DNA viruses is notably lower [80]. The accordion model of continuous alternate deletion and duplication events may explain how a dsDNA virus adapts rapidly to a new environment or host [27, 28]. However, even though some NCLDV genomes contain a high number of duplicated genes (Table 3, Fig. S8), duplications affect only a small proportion of protein clusters.

**Table 3.** Protein clusters that have the highest copy number in widest range of NCLDV genomes

Protein cluster	Pfam protein families (no. of cluster members) identified among members of a cluster and their description	No. of genomes in which protein clusters have >5 members/total no. of genomes in which protein cluster is present	Copy no. (>5)	NCBI assembly accession (genome size as kb)
Cluster_1	**PF00023** (61) Ankyrin repeat **PF00646** (30) Fbox domain **PF03158** (8) Multigene family 530 protein **PF09372** (105) PRANC domain **PF12796** (371) Ankyrin repeats **PF12937** (94) F-box-like **PF13606** (16) Ankyrin repeat **PF13637** (50) Ankyrin repeats **PF13639** (1) Ring finger domain **PF13857** (11) Ankyrin repeats	28 (6–307 members)/59	9 116 98 131 16 132 217 307 8 8 10 50 26 14 30 34 6 11 15 7 9 13 11 7 7 7 7 7	GCF_000858485.1 (170) GCF_000888735.1 (1181) GCF_000904035.1 (1021) GCF_000893915.1 (1259) GCF_001292995.1 (651) GCF_000911655.1 (1908) GCF_000928575.1 (2243) GCF_000911955.1 (2473) GCF_000847045.1 (330) GCF_000871245.1 (344) GCF_000839765.1 (336) GCF_000841685.1 (359) GCF_000922075.1 (282) GCF_001431935.1 (189) GCF_000838605.1 (289) GCF_000923135.1 (307) GCF_000892975.1 (176) GCF_000839105.1 (206) GCF_000839185.1 (224) GCF_000841905.1 (210) GCF_000857045.1 (197) GCF_001029045.1 (215) GCF_000869985.1 (198) GCF_000860085.1 (195) GCF_000859885.1 (186) GCF_000844045.1 (134) GCF_000930695.1 (140) GCF_000886295.1 (145)
Cluster_2	**PF01541** (6) GIY-YIG catalytic domain **PF02498** (60) BRO family, N-terminal domain **PF02796** (7) Helix-turn-helix domain of resolvase **PF03288** (6) Poxvirus D5 protein-like **PF04218** (2) CENP-B N-terminal DNA-binding domain **PF04383** (145) KilA-N domain **PF04480** (1) Protein of unknown function **PF10544** (37) T5orf172 domain **PF10553** (65) MSV199 domain **PF12299** (61) Protein of unknown function **PF13639** (16) Ring finger domain	15 (6–47 members)/49	7 12 14 18 15 13 18 16 16 6 28 13 16 47 27	GCF_000871485.1 (186) GCF_000881595.1 (119) GCF_000838105.1 (212) GCF_000923155.1 (220) GCF_000916235.1 (196) GCF_000909775.1 (198) GCF_000915575.1 (199) GCF_000914535.1 (205) GCF_000891235.1 (206) GCF_000918955.1 (163) GCF_000916855.1 (246) GCF_000837185.1 (232) GCF_000427135.1 (229) GCF_000427115.1 (308) GCF_000427175.1 (283)
Cluster_7	**PF04451** (85) Large eukaryotic DNA virus major capsid protein **PF16903** (81) Major capsid protein N-terminus	8 (7-8 members)/59	8 7 8 8 8 8 8 8	GCF_000872425.2 (187) GCF_000889515.1 (199) GCF_000890375.1 (184) GCF_000888835.1 (194) GCF_001399285.1 (196) GCF_001399225.1 (182) GCF_000885975.1 (192) GCF_000887855.1 (184)

Vaccinia virus (a member of the *Poxviridae*) has been shown to have duplicate genes that face a sudden positive selection pressure [27]. The poxvirus protein K3L is needed for inhibiting host protein kinase R that is part of the innate defence machinery against viruses [81]. The gene copy number of K3L quickly expanded when selective pressure on the virus was increased by experimentally mutating another protein kinase R inhibitor, the poxvirus protein E3L, making it non-functional [27]. In some cases, the increased copy number contributed to increase the genome size by 7–10 % within 10 passages [27]. When the pressure was relaxed the copy number of K3L was reduced [27]. Interestingly, these results are comparable to those of Boyer *et al*., in which the mimivirus genome shrunk by 16 % after growing *Acanthamoeba castellanii* in an axenic environment [59]. These observations indicate that NCLDVs can adapt to environmental changes by rapid duplications and deletions of genes.

Notably, the copy number of gene families can vary dramatically between NCLDV species (Table 3). Genome comparisons of members of *Mimiviridae* (1181–1245 kb) and chlorellavirus (a member of *Phycodnaviridae*; genome 340–369 kb) indicated the higher importance of gene duplications and losses events in contributing to their genome variations, whereas NCLDVs infecting algae of the species *Micromonas* (member of *Phycodnaviridae*; genomes 173–187 kb) and *Ostreococcus* (a member of *Phycodnaviridae*; genomes 184–194 kb) were mainly affected by LGT [28].

The highest copy number of a protein cluster in NCLDVs varies notably and it correlates with the genome size (Figs S8 and S11). The protein cluster that most often gives the highest copy number within an NCLDV genome is cluster 1, which contains repeats of ankyrin-domains (Table 3). They are especially numerous in the largest amoeba-infecting viruses but also poxviruses (Table 3). Ankyrin domains are very common among eukaryotes and they are known to mediate protein–protein interactions in diverse contexts such as cell signalling and differentiation [82]. Poxviruses may use ankyrin-domain- containing proteins for the suppression of several cellular antiviral pathways [83, 84]. Interestingly, the copy number of genes encoding protein containing the ankyrin domain may change quickly, as ankyrin repeats were largely deleted in mimivirus, when its amoebal host was grown in axenic culture [48], suggesting that these proteins are needed for virus survival in a phagocytosing host cell. Cluster 2 gives the highest copy numbers in insect-infecting NCLDVs, whereas prasinoviruses have multiple copies of major capsid protein (Table 3, Fig. S9). Notably, the high copy numbers are independent of the average copy number or proportion of multicopy protein clusters in the NCLDV genome, indicating that duplication pressure affects mainly specific protein clusters (Fig. S8). Despite the fact that duplication and deletion events have notable effects on NCLDV genome sizes [27, 59], this does not totally explain the extremely large genomes of both pandoraviruses and mimiviruses, as the proportion of copied ORFs in these genomes does not correlate with the genome size (Fig. S8b) as *de novo* gene formation and LGT also contribute to their large genome sizes and coding capacity.

Recently, *de novo* gene creations have been proposed to explain the high number of strain-specific genes among pandoravirus genomes [33]. There are two main hypotheses about the mechanisms for *de novo* gene creation: (1) the intergenic region acquires transcription before evolving an ORF or (2) vice versa [33]. Over 80 % of pandoravirus genomes have been transcribed, whereas only 62–68.2 % of transcripts were shown to be translated, consistent with the first mechanism [33]. The *de novo* gene creation hypothesis was supported by the notion that the species-specific genes in pandoraviruses were different from core genes based on properties such as G+C content and smaller length. However, only two translated species-specific ORFs have been observed in intergenic regions of two closely related species [33].

#### Insertions, deletions and duplications are localized to the end of the genome and involve genes needed for host–virus interactions

The ends of the genomes among linear NCLDVs have been reported to be less conserved and to be more prone to LGT and genes losses, and to be under diversifying selection. For example, the conserved genes among *Poxviridae* are mapped to locations in the middle of the vaccinia virus genome [85]. These conserved proteins were responsible for the core functions of replication, transcription, virion morphogenesis, virion assembly and maturation [85]. Moreover, chordopoxvirus genes with evidence for the strongest diversification pressure were noted to locate at the end of the genome [86]. These proteins were shown in particular to mediate host–pathogen interactions, modulating host range and virulence [86]. In addition, experimental studies have shown that, if E3L was mutated in Vaccinia virus, replacing duplications of the K3L gene appeared at the end of genomes [27]. In contrast, more broadly conserved orthologous gene families within species of the family *Mimiviridae* are located in the centre of the genomes, whereas their duplicated genes were located at the end of their respective genomes [87]. The extremities of NCLDV genomes can also contain members of some conserved gene families, but their synteny and orientation are typically not conserved [87]. Such an unequal distribution of core genes is also observed among NCLDVs with a circular genome [88]. Among the members of *Marseilleviridae*, the core gene regions are concentrated in a region covering one-third of the genome, in which genome rearrangements are nearly absent and primary sequence slightly more conserved [88].

Many NCLDVs tolerate notably large insertions and deletions at the tips of their genome. For example, in chlorellaviruses 27–45 kb deletions and 22.2 kb insertions at the left end of the genome have been described [30, 89–91], and 90.5 kb and 95.6 kb insertions described in mimiviruses, when grown in axenic amoeba culture [59]. Notably, the inserted and deleted regions in chlorellavirus were encoding in particular glycoproteins [30, 89]. These glycoproteins in the 22.2 kb insertion were expressed during the late phase of the infection and they were also included in the virion, suggesting their importance in structural variation of the virus [92]. In human pathogens, glycosylation of viral proteins can contribute to virion binding to the host receptor, protein folding as well as hiding from neutralizing antibodies [93].

### Conclusions

NCLDVs are an extraordinarily diverse group of viruses with a broad host range. Due to their unique genome characteristics, including the presence of translational cellular genes in the largest NCLDVs, it was suggested that these unusually complex viral genomes were derived from an unknown fourth domain of cellular life. The sharing of a number of gene families across a broad range of NCLDV lineages also suggested that these viruses are monophyletic. However, phylogenies inferred under the best-fitting models for the translational cellular genes indicate multiple independent acquisitions from various cellular lineages. Notably, there is also only a single viral protein family that is shared across all NCLDV lineages, and among the five most broadly distributed protein families only four support the monophyly of NCLDVs, including three viral protein families and one protein family shared with eukaryotes. Moreover, recently described new viruses suggest that NCLDV core genes can be present sporadically in non-NCLDV virus genomes, blurring further the boundary between NCLDVs and non-NCLDV viruses, possibly shared though LGT events. Viral genome gigantism has probably evolved independently at least twice, and our shared protein content analyses and individual gene phylogenies further support this by indicating that (i) mimiviruses are related to the members of the *Phycodnaviridae* characterized by smaller-genomes and (ii) the pandoviruses, with the largest known NCLDVs genomes, are more closely related to the mollivirus characterized by a relatively smaller genome. These different considerations provide support for neither an origin for the NCLDVs from a fourth domain of life nor strong evidence for a coherent monophyletic NCLDV lineage. Notably, virus genome size seems to enlarge or shrink depending on environmental factors, such as eukaryote(host)–bacteria–virus interactions. The observed dramatic genome size variations involve several mechanisms, including gene duplications and deletions, LGTs and *de novo* gene formation, and these events can have a significant impact in a short time frame as demonstrated through experimentation with different NCLDVs in various hosts. The majority of these changes are concentrated on the end of linear NCLDV genomes, where proteins for host–virus interactions are typically located. A biased distribution of the fastest evolving genome segments was also observed in viruses with circular genomes. In addition, nearly 60 LGTs between insect-infecting NCLDVs, and NCLDVs and unrelated insect-infecting viruses indicate how LGTs could contribute to NCLDV adaptations to their insect hosts. The discovery of the fascinating NCLDVs was sensational and highlighted an important gap in our knowledge of the virome of planet Earth, and recent metagenomic surveys have also further established their global distribution and high abundance in various environments. Although the hypothesis for their cellular origin was exciting, detailed analyses of protein families established that their origins are still a mystery, and like all other viral lineages we cannot establish whether they are derived from an ancestral cellular life form, originated from selfish elements (e.g. transposable elements) or even from the primordial soup from which all terrestrial cellular life forms are thought to have originated, possibly involving pre-cellular primordial replicators [94]. In addition, recently identified substantial NCLDV-to-eukaryote LGTs among the genomes of some green algae imply that these viruses may impact their host genome evolution in multiple ways in some lineages and by doing so could eventually contribute to novel cellular protein-coding genes. We are still in the early days of the study of NCLDVs. From what has been discovered so far about NCLDVs strongly suggests that we can expect further exciting new discoveries about their biology and the complex and intricate relationships between viruses and their eukaryotic hosts.

## Supplementary Data

Supplementary material 1
