# Supplementary material for: The genomes of nucleocytoplasmic large DNA viruses: viral evolution writ large
Source: Microb Genom. 2021 Sep 20;7(9):000649. doi: 10.1099/mgen.0.000649 (PMC8715426; doi:10.1099/mgen.0.000649)
Supplement: Supplementary material 1 [file mgen-7-0649-s001.pdf]

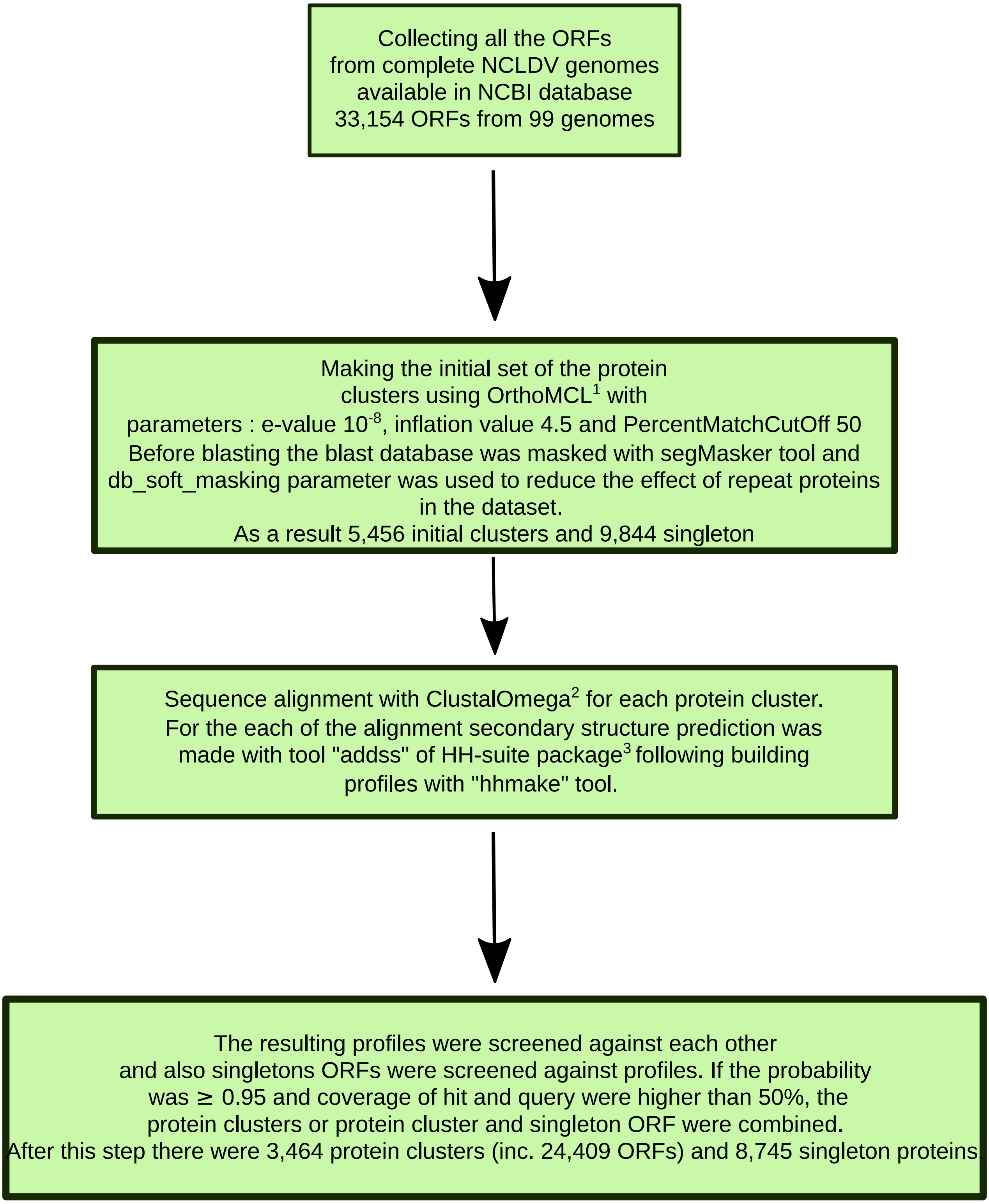

Supplementary Figure S1. Workchart for building of the protein clusters.

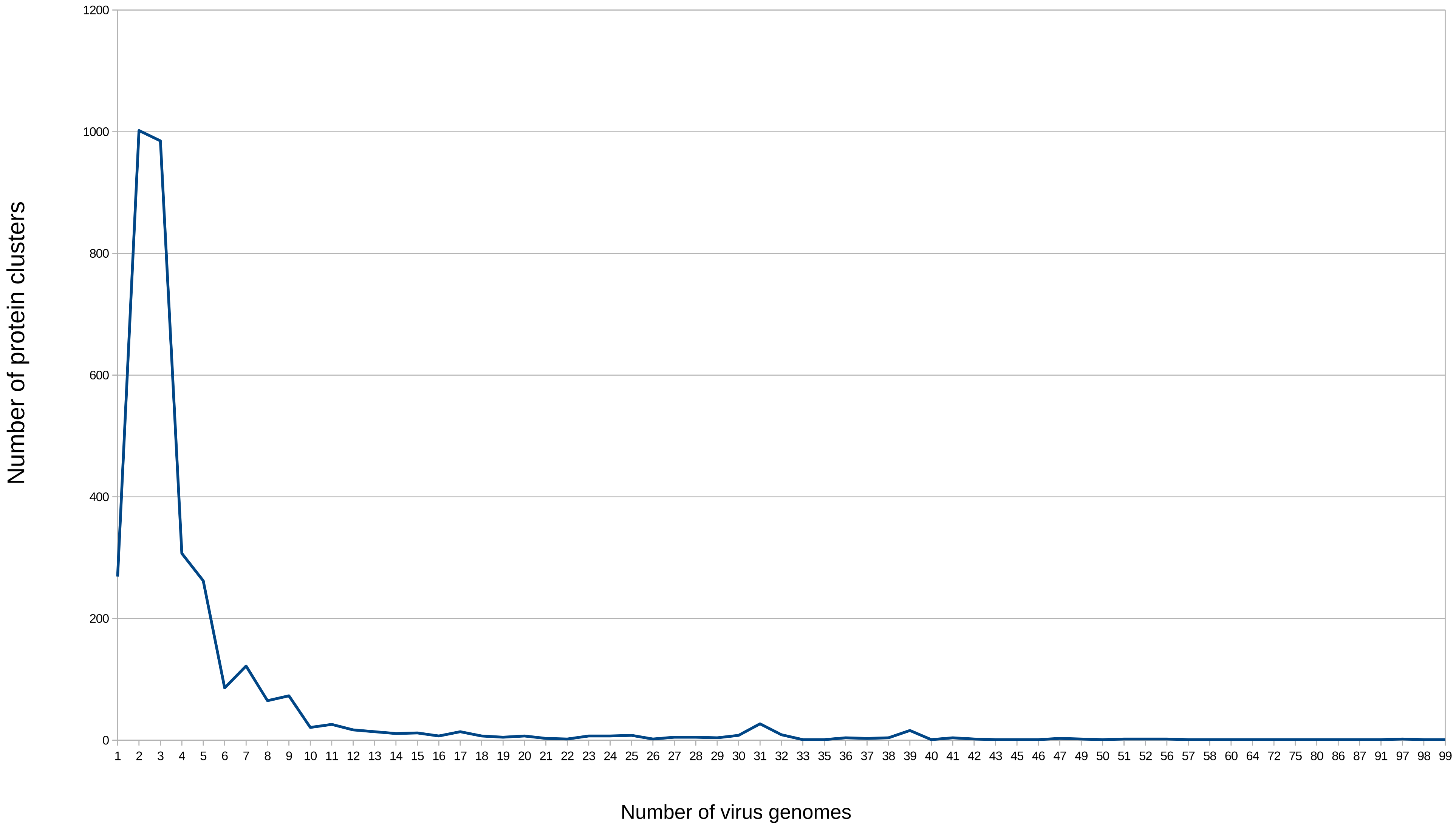

Supplementary Figure S2. Number of NCLDV genomes sharing a protein cluster. The graph shows how many protein clusters are shared by a certain number of NCLDV genomes.

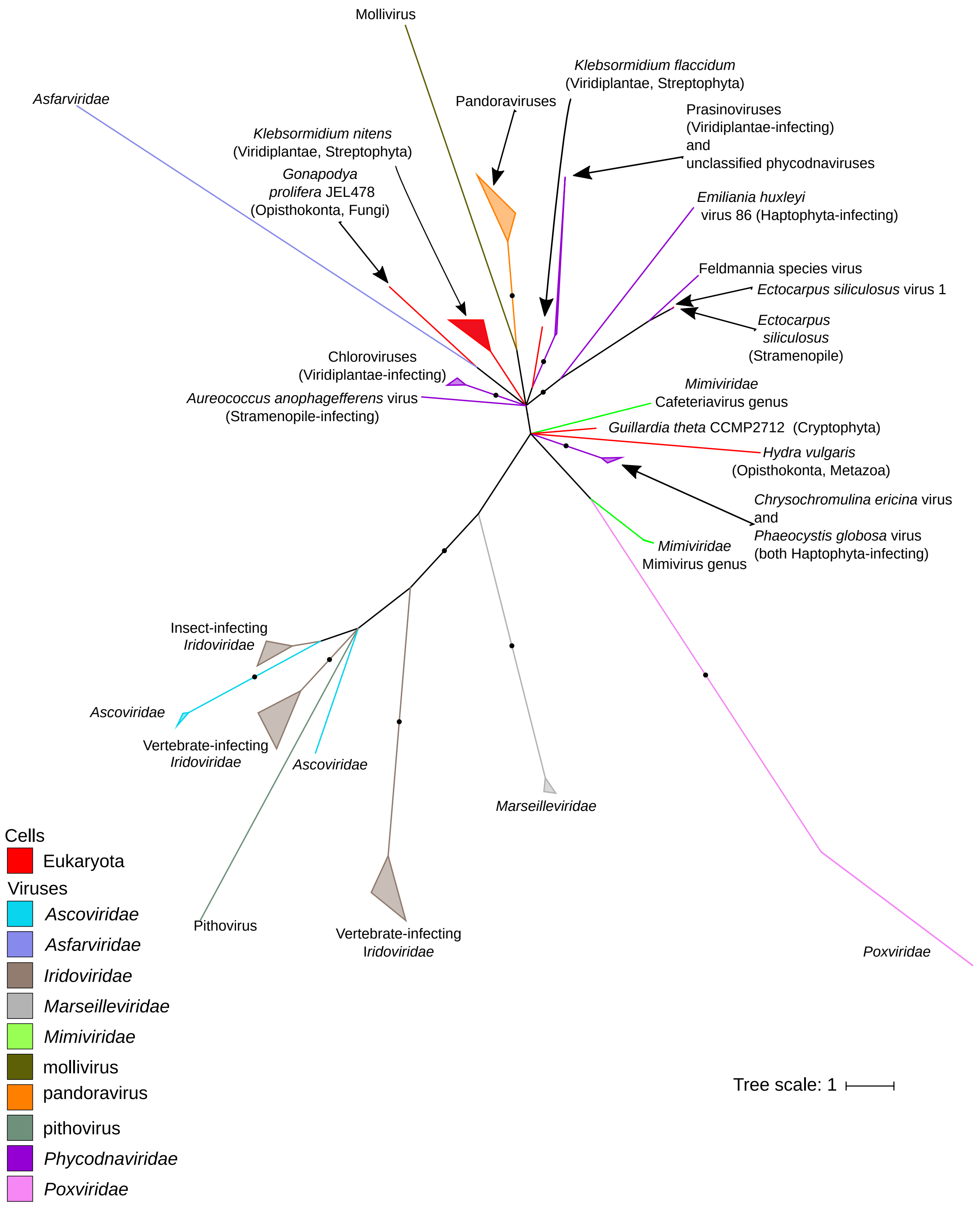

Supplementary Figure S3. Phylogeny of Poxvirus late transcription factor VLTF3-like (Pfamid: PF04947) open-reading frames. The tree is based on the amino acid sequences representing the protein cluster 1 in the Figure 1 (Identifier in the data set: cluster_11) and the best five blastp hits outside NCLDVs for each NCLDV sequence (e-value < 10^{-5}). The screen was done against NCBI non-redundant protein database (downloaded on the 18th of July 2016). The proteins which length was less than 80 % from the average length of the VLTF3-like domain in the dataset, were removed. The eukaryotic sequences were checked for contaminants. The scaffold in which corresponding eukaryotic gene located, had to contain an ORF that received the best hit from an eukaryotic ORF in another scaffold. The alignment was performed using MAFFT v7 L-INS-i[4]. The alignment was trimmed using Trimal with "automated1" settings[5]. Sites present only in a single sequence, were removed. The length of final alignment was 150 sites for 110 sequences. The tree was inferred under LG+C60[6,7] model in PhyloBayes[8]. The posterior probabilities that are higher than 0.95 are indicated with black dots. iTOL[9] was used for visualization of the phylogeny.

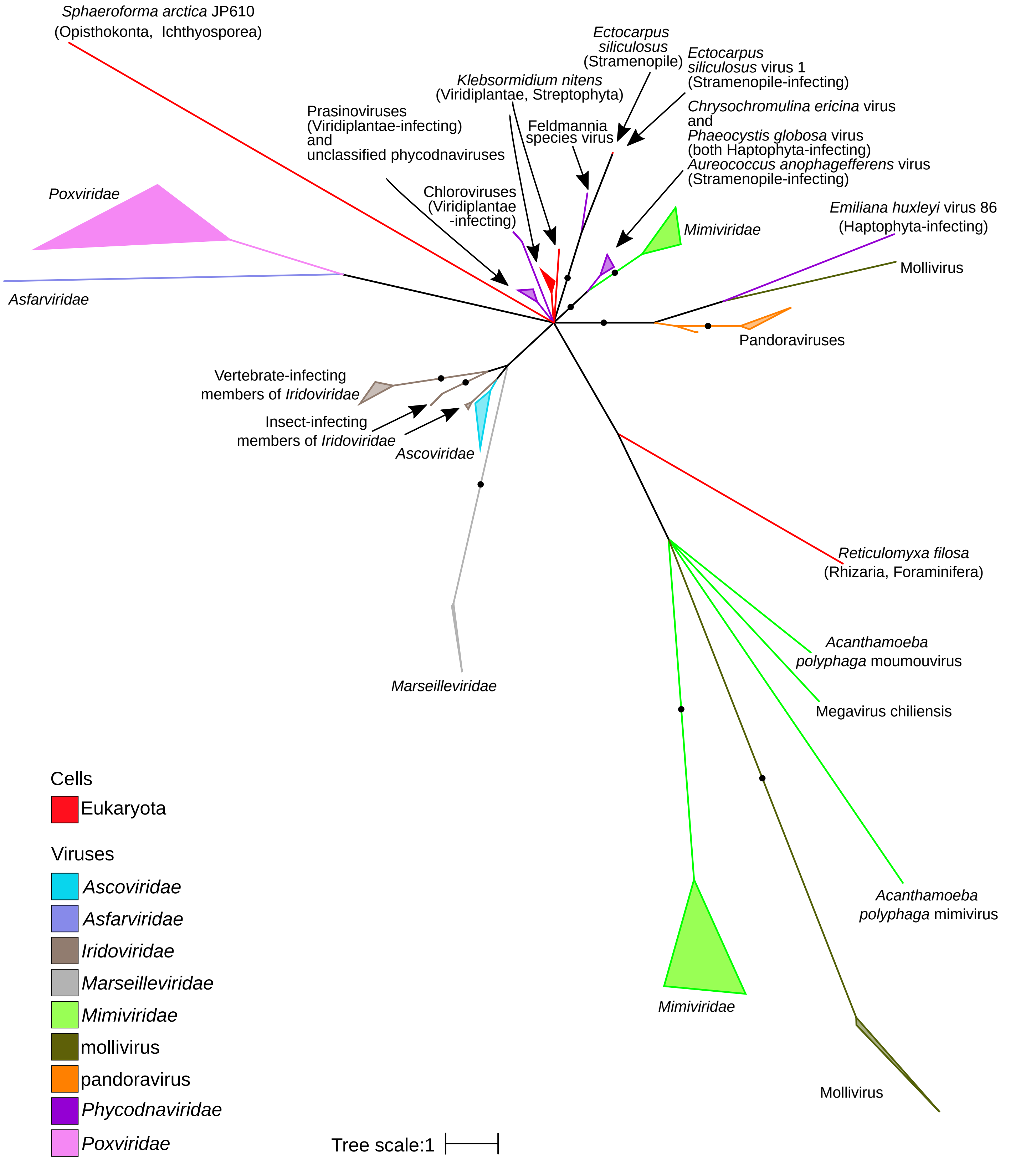

Supplementary Figure S4. Phylogenetic tree for Poxvirus A32 protein (packaging ATPase) (Pfam: PF04665). The tree is based on the amino acid sequences representing the protein cluster 2 in the Figure 1 (identifier in the data set: Cluster_9). The tree was processed as described Supplementary Figure S3. The alignment was checked and sites conserved less than 30 % sites were removed. The lengths of the final alignments were 129 sites for 117 sequences.

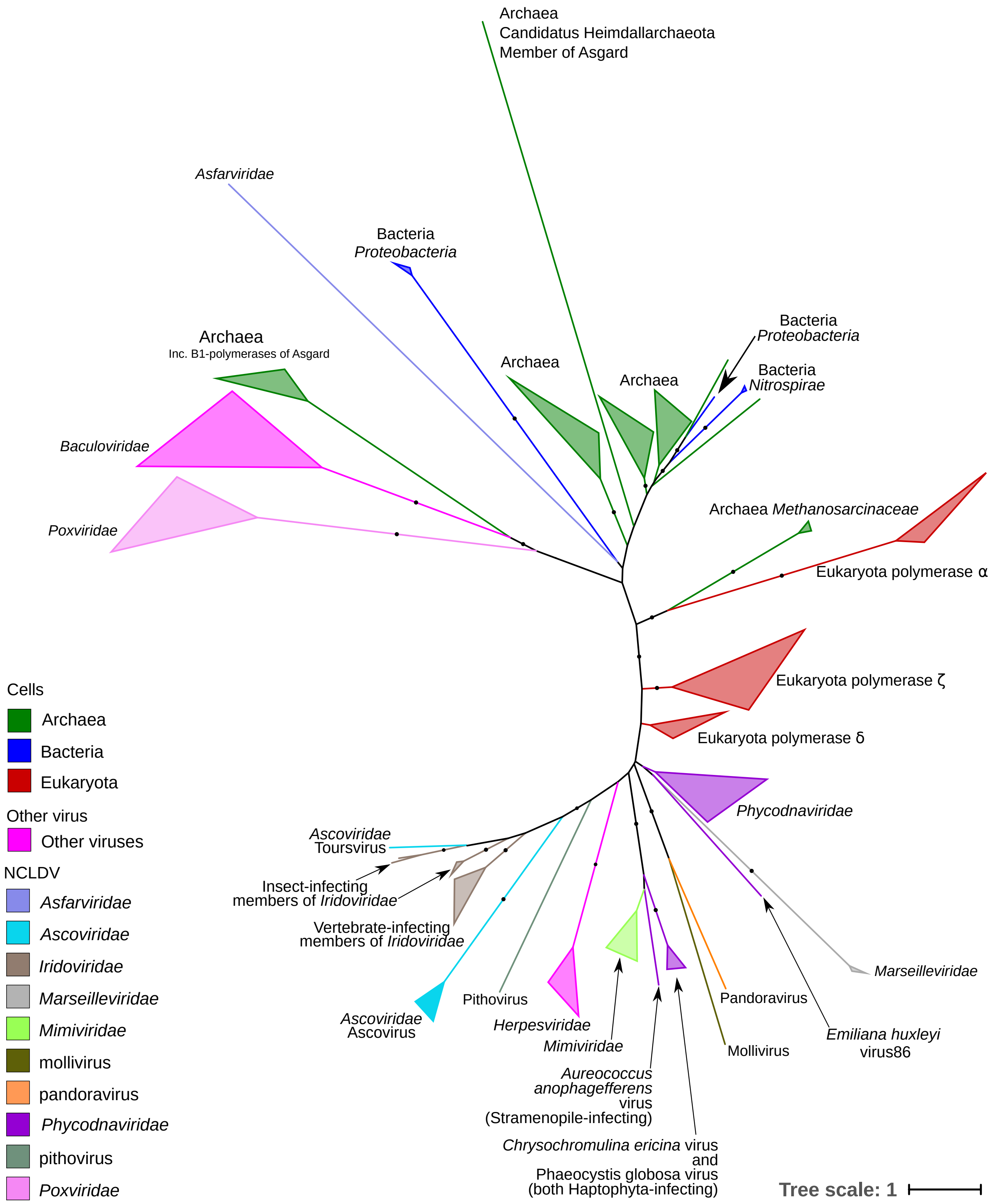

Supplementary Figure S5. Phylogenetic tree polymerase B family DNA polymerases (Pfamid: PF00136). The tree is based on the amino acid sequences representing the protein cluster 4 in the Figure 1 (identifier in the data set: cluster_12), and the best five pblast hits outside NCLDVs for each NCLDV sequence (e-value < 10^{-5}). The screen was done against NCBI non-redundant protein database (downloaded on the 18th of July 2016). To expand the selection of sequences, more distant members of DNA polymerase were considered including representatives from all archaeal phylums as well as eukaryotic polymerases δ , ζ and α. Eukaryotic polymerase ε and phage polymerases were left out due to short sequence similarity (they shortened the alignment from 334 sites to 192 sites). As polymerase proteins contain multiple domains that may disturb the alignment, the polymerase domains were identified using Interproscan[10], and to confirm the completeness of the domain, 100 amino acids were added at the both ends. These extracted sequences were processed and aligned as in the Supplementary Figure S3. The tree was inferred under LG+C60[6,7] model in IQ-TREE[11] with ultrafast bootstrap[12], because the tree did not converged in PhyloBayes. The ultrafast boostrap values that are higher than 0.95 are indicated with black dots.

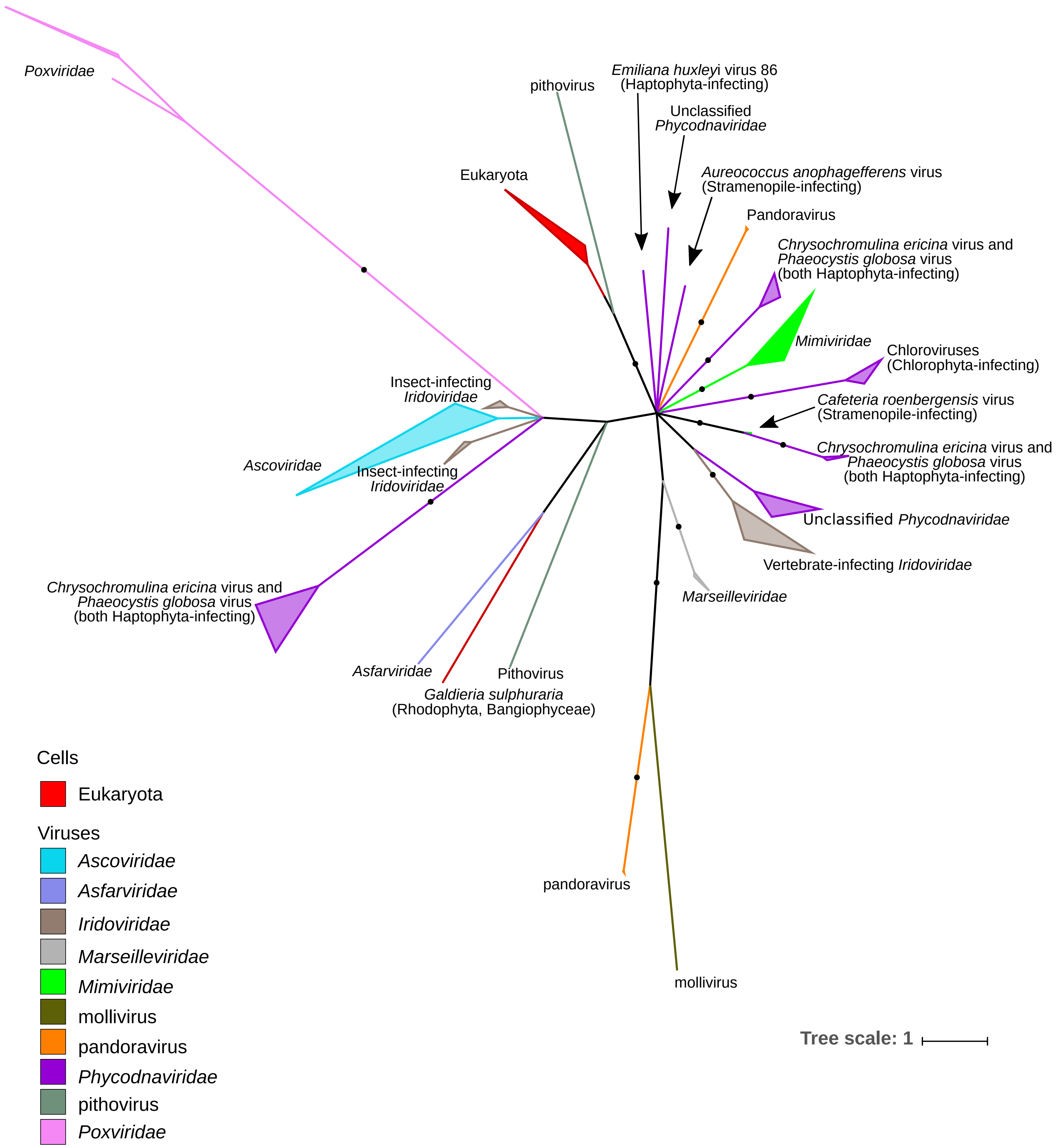

Supplementary Figure S6. Phylogenetic tree for disulfide (thiol) oxidoreductase, Erv1/Alr family (Pfam: PF04777). The tree is based on the amino acid sequences representing the protein cluster 6 in the Figure 1 (identifier in the dataset: cluster_13). The sequences were selected, processed and aligned as in Supplementary Figure S3. Eukaryotic sequences locating in independent branches separately from the other eukaryotic sequences were screened for potential contaminants. The length of the final alignment was 80 sites for 261 sequences.

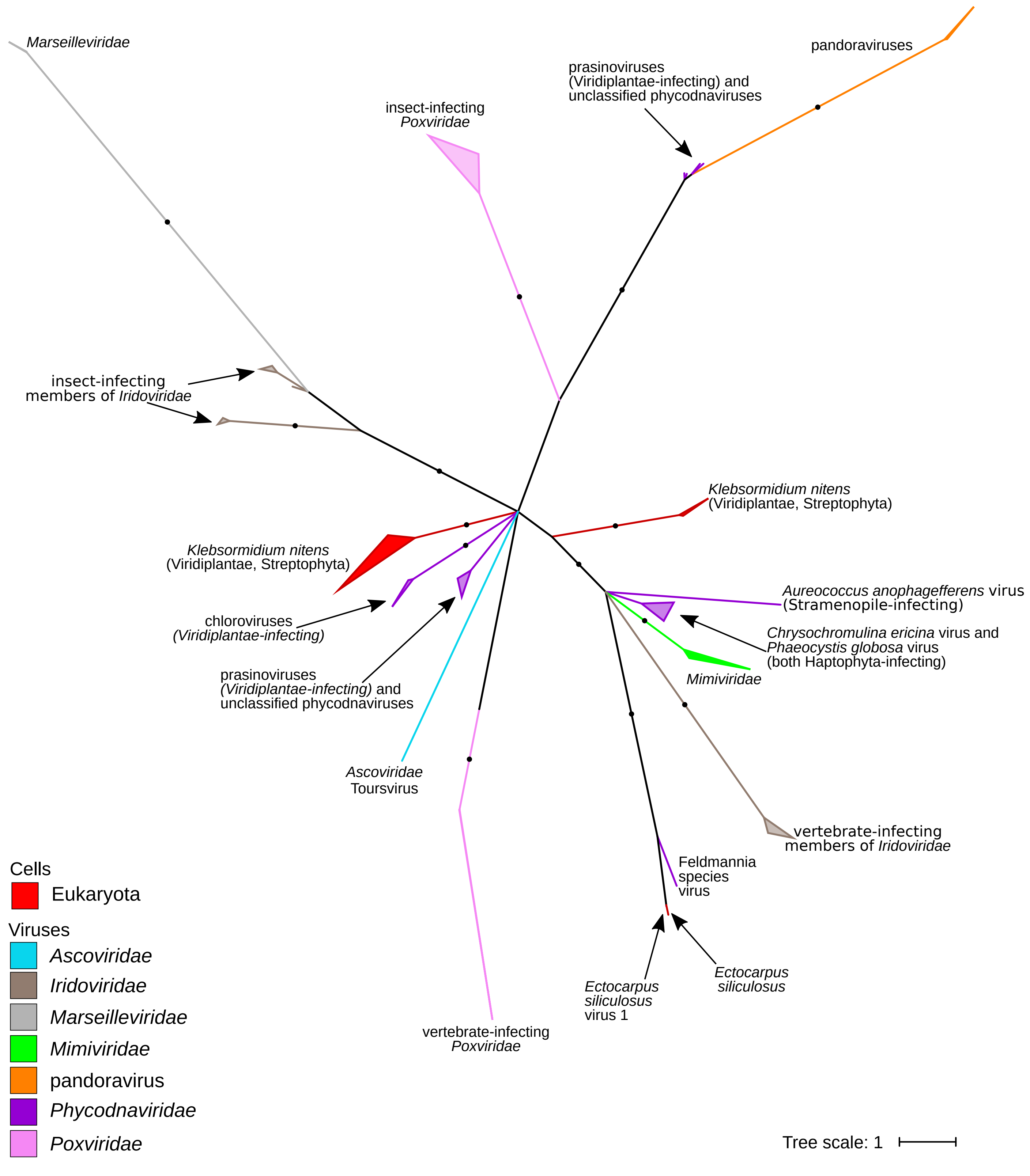

Supplementary Figure S7. Phylogenetic tree for Poxvirus A22 protein (Pfamid: PF04848). The tree is based on the amino acid sequences representing the protein cluster 7 in the Figure 1 (identifier in the dataset: cluster_15). The sequences were selected, processed and aligned and phylogeny made as in Supplementary Figure S3. The length of the final alignment was 98 sites for 95 sequences.

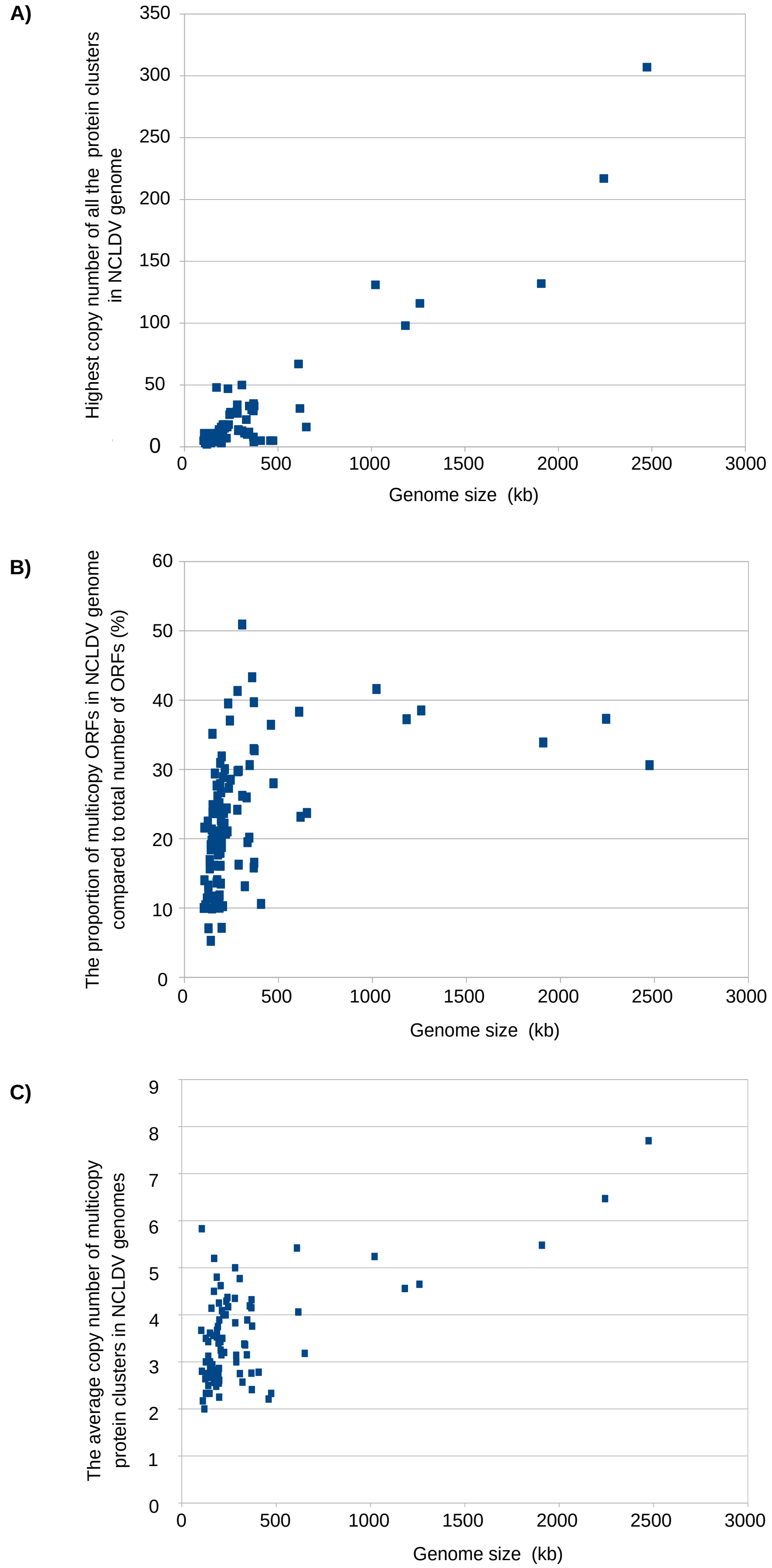

Supplementary Figure S8. Copy number variation in NCLDV genomes. The copy number for each protein cluster within each NCLDV genome was calculated. From these data three scatter plots were generated. The highest copy number of all protein clusters in each NCLDV genome compared to the NCLDV genome size (A), the proportion of multicopying ORFs (>2) within a NCLDV genome compared to the NCLDV genome size (B). The average copy number of protein clusters (single copy proteins were left out) compared to the NCLDV genome size (C).

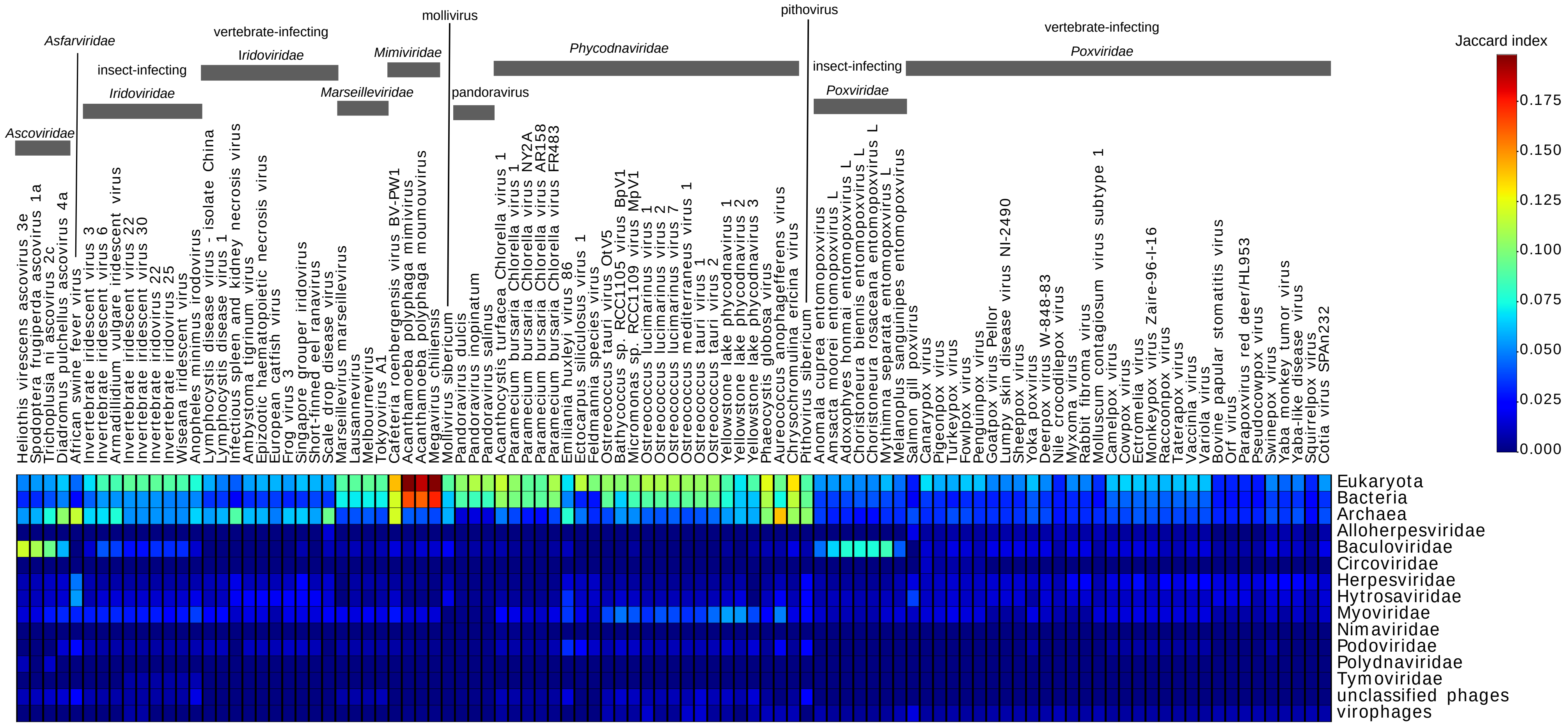

Supplementary Figure S9. The Jaccard indices indicating proportion of shared protein clusters between NCLDVs and cellular life or other viruses. The Jaccard index describes the size of intersections between two groups. The higher the Jaccard index is the bigger is the proportion of the shared protein clusters. The Jaccard indices are calculated from bipartite network in Figure 3. The protein cluster is considered to be present in cellular life or other viruses if at least one protein cluster member receives a protein blast hit outside NCLDVs from cellular life or other virus. The screen was done against NCBI non-redundant protein database that was downloaded on the 18th of July 2016. NCLDV species and families are shown on the top of the heatmap and cellular life or virus taxonomy on the right. The Jaccard index scale is presented on the right.

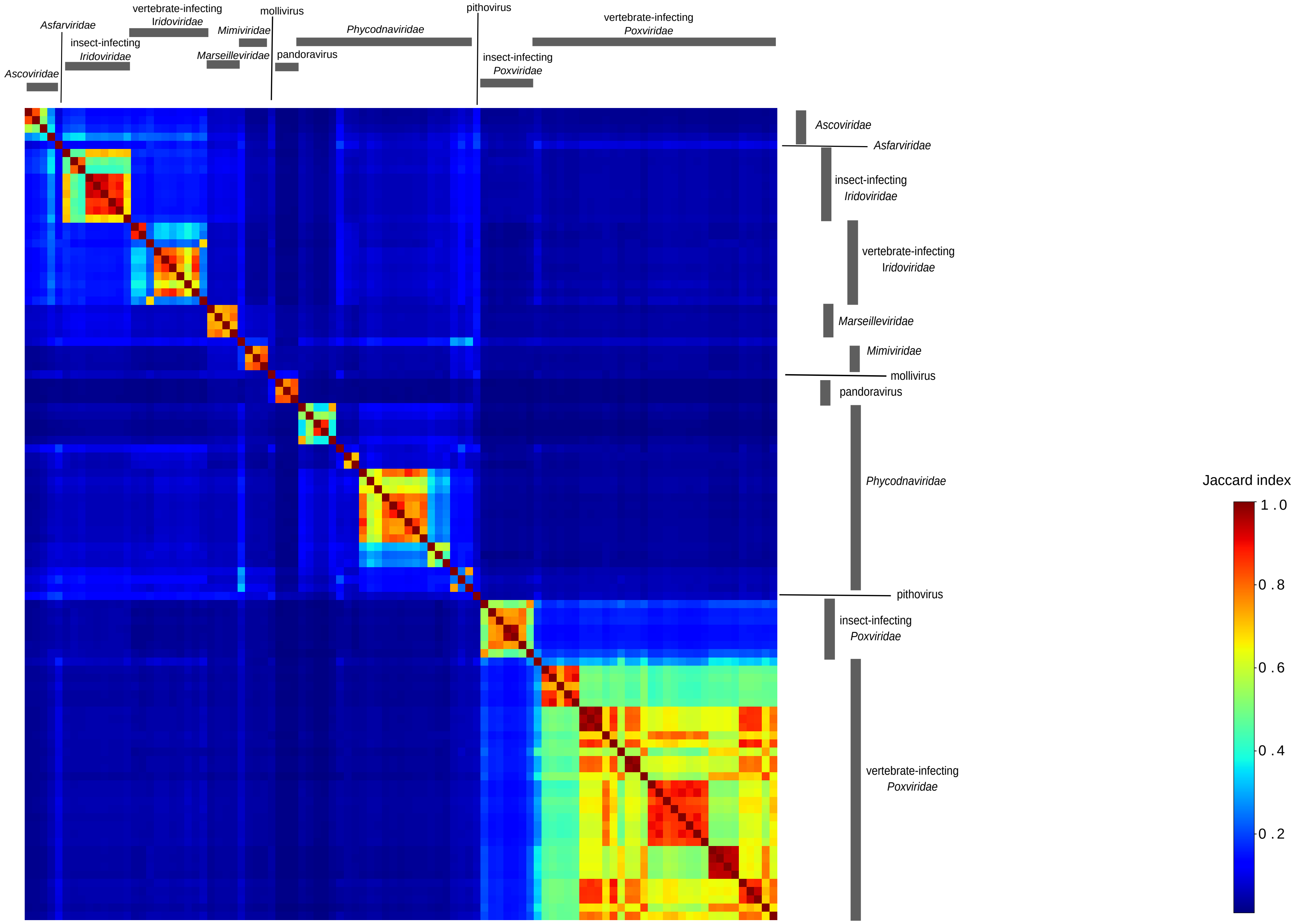

Supplementary Figure S10. Heatmap of the Jaccard indeces indicating the proportion of shared protein clusters between the NCLDV genomes. The Jaccard index describes the size of intersections between two groups. The higher the Jaccard index is, the bigger is the proportion of the shared protein clusters. The Jaccard indices are calculated from the bipartite network in Figure 3. The screen was done against NCBI non-redundant protein database (downloaded on the 18th of July 2016). NCLDV families or groups are indicated with bars and lines on the right side and top of the heatmap. The Jaccard index scale is presented on the right.

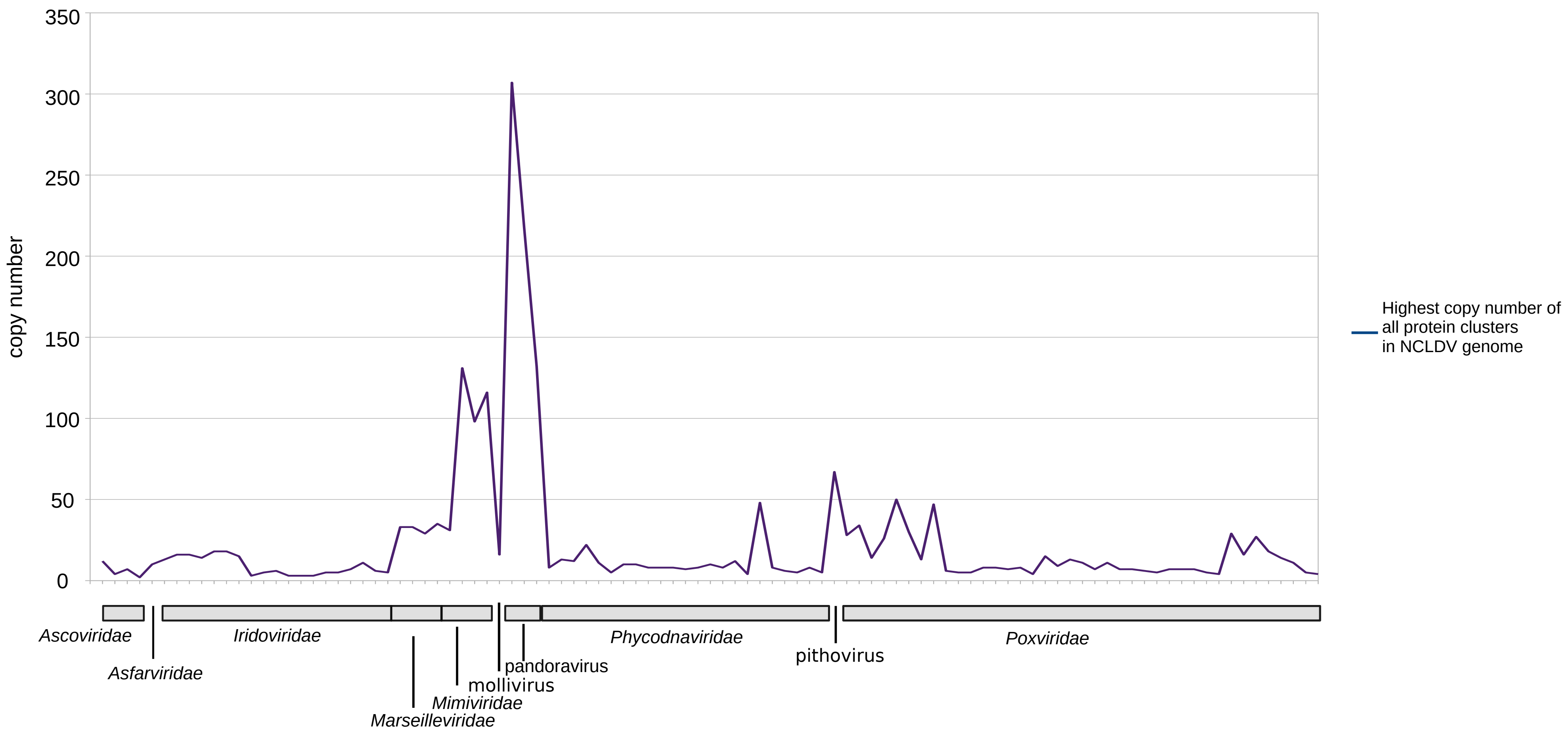

Supplementary Figure S11. The highest copy number of all the protein clusters in each NCLDV genome. The copy number of each protein cluster within each NCLDV genome was counted. The highest copy number for each genome is plotted on the graph. The NCLDV lineages are presented on the x axis.

Supplementary Table S1. The NCLDV genome assemblies used in the study

Number	Family/genus	Species	Accession (NCBI assembly)	Reference
1	*Ascoviridae*	Heliothis virescens ascovirus 3e	GCF_000871485.1	13
2	*Ascoviridae*	Spodoptera frugiperda ascovirus 1a	GCF_000867605.1	14
3	*Ascoviridae*	Trichoplusia ni ascovirus 2c	GCF_000868565.1	15
4	*Ascoviridae*	Diadromus pulchellus ascovirus 4a	GCF_000881595.1	16
5	*Asfarviridae*	African swine fever virus	GCF_000858485.1	17
6	*Iridoviridae*	Invertebrate iridescent virus 3	GCF_000869125.1	18
7	*Iridoviridae*	Invertebrate iridescent virus 6	GCF_000838105.1	19
8	*Iridoviridae*	Armadillidium vulgare iridescent virus	GCF_000923155.1	20
9	*Iridoviridae*	Invertebrate iridescent virus 22	GCF_000916235.1	21
10	*Iridoviridae*	Invertebrate iridescent virus 30	GCF_000915575.1	21
11	*Iridoviridae*	Invertebrate iridovirus 22	GCF_000909775.1	22
12	*Iridoviridae*	Invertebrate iridovirus 25	GCF_000914535.1	23
13	*Iridoviridae*	Wiseana iridescent virus	GCF_000891235.1	24
14	*Iridoviridae*	Anopheles minimus irodovirus	GCF_000918955.1	25
15	*Iridoviridae*	Lymphocystis disease virus - isolate China	GCF_000844885.1	26
16	*Iridoviridae*	Lymphocystis disease virus 1	GCF_000839605.1	27
17	*Iridoviridae*	Infectious spleen and kidney necrosis virus	GCF_000848865.1	28
18	*Iridoviridae*	Ambystoma tigrinum stebbensi virus	GCF_000841005.1	29
19	*Iridoviridae*	Epizootic haematopoietic necrosis virus	GCF_001448375.1	30
20	*Iridoviridae*	European catfish virus	GCF_000897115.1	31
21	*Iridoviridae*	Frog virus 3	GCF_000844425.1	32
22	*Iridoviridae*	Singapore grouper iridovirus	GCF_000846905.1	33
23	*Iridoviridae*	Short-finned eel ranavirus	GCF_001678255.1	34
24	*Iridoviridae*	Scale drop disease virus	GCF_001274405.1	35
25	*Marseilleviridae*	Marseillevirus marseillevirus	GCF_000887095.1	26
26	*Marseilleviridae*	Lausannevirus	GCF_000893455.1	37
27	*Marseilleviridae*	Melbournevirus	GCF_000924835.1	38
28	*Marseilleviridae*	Tokyovirus A1	GCF_001654305.1	39
29	*Mimiviridae*	Cafeteria roenbergensis virus BV-PW1	GCF_000889395.1	40
30	*Mimiviridae*	Acanthamoeba polyphaga mimivirus	GCF_000888735.1	41
31	*Mimiviridae*	Acanthamoeba polyphaga moumouvirus	GCF_000904035.1	42
32	*Mimiviridae*	Megavirus chiliensis	GCF_000893915.1	43
33	Mollivirus	Mollivirus sibericum	GCF_001292995.1	44
34	Pandoravirus	Pandoravirus dulcis	GCF_000911655.1	45
35	Pandoravirus	Pandoravirus inopinatum	GCF_000928575.1	46
36	Pandoravirus	Pandoravirus salinus	GCF_000911955.1	45
37	*Phycodnaviridae*	Acanthocystis turfacea Chlorella virus 1	GCF_000869685.1	47
38	*Phycodnaviridae*	Paramecium bursaria Chlorella virus 1	GCF_000847045.1	48
39	*Phycodnaviridae*	Paramecium bursaria Chlorella virus NY2A	GCF_000873685.1	49
40	*Phycodnaviridae*	Paramecium bursaria Chlorella virus AR158	GCF_000871245.1	49
41	*Phycodnaviridae*	Paramecium bursaria Chlorella virus FR483	GCF_000867825.1	50
42	*Phycodnaviridae*	Emiliania huxleyi virus 86	GCF_000865825.1	51
43	*Phycodnaviridae*	Ectocarpus siliculosus virus 1	GCF_000839765.1	52
44	*Phycodnaviridae*	Feldmannia species virus	GCF_000874805.1	53
45	*Phycodnaviridae*	Ostreococcus tauri virus OtV5	GCF_000872425.2	54
46	*Phycodnaviridae*	Bathycoccus sp. RCC1105 virus BpV1	GCF_000889515.1	55
47	*Phycodnaviridae*	Micromonas sp. RCC1109 virus MpV1	GCF_000890375.1	55
48	*Phycodnaviridae*	Ostreococcus lucimarinus virus 1	GCF_000888835.1	55
49	*Phycodnaviridae*	Ostreococcus lucimarinus virus 2	GCF_001399285.1	56
50	*Phycodnaviridae*	Ostreococcus lucimarinus virus 7	GCF_001399225.1	56
51	*Phycodnaviridae*	Ostreococcus mediterraneus virus 1	GCF_001399265.1	56
52	*Phycodnaviridae*	Ostreococcus tauri virus 1	GCF_000885975.1	57
53	*Phycodnaviridae*	Ostreococcus tauri virus 2	GCF_000887855.1	58
54	*Phycodnaviridae*	Yellowstone lake phycodnavirus 1	GCF_001430655.1	59
55	*Phycodnaviridae*	Yellowstone lake phycodnavirus 2	GCF_001430455.1	59
56	*Phycodnaviridae*	Yellowstone lake phycodnavirus 3	GCF_001430035.1	59
57	*Phycodnaviridae*	Phaeocystis globosa virus	GCF_000907415.1	60
58	*Phycodnaviridae*	Aureococcus anophagefferens virus	GCF_000922335.1	61
59	*Phycodnaviridae*	Chrysochromulina ericina virus	GCF_001399245.1	62
60	Pithovirus	Pithovirus sibericum	GCF_000916835.1	63
61	*Poxviridae*	Anomala cuprea entomopoxvirus	GCF_000865825.1	64
62	*Poxviridae*	Amsacta moorei entomopoxvirus	GCF_000837185.1	65
63	*Poxviridae*	Adoxophyes honmai entomopoxvirus 'L'	GCF_000427135.1	66
64	*Poxviridae*	Choristoneura biennis entomopoxvirus	GCF_000427115.1	66
65	*Poxviridae*	Choristoneura rosaceana entomopoxvirus 'L'	GCF_000427175.1	66
66	*Poxviridae*	Mythimna separata entomopoxvirus 'L'	GCF_000427155.1	66
67	*Poxviridae*	Melanoplus sanguinipes entomopoxvirus	GCF_000838565.1	67
68	*Poxviridae*	Salmon gill poxvirus	GCF_001271235.1	68
69	*Poxviridae*	Canarypox virus	GCF_000841685.1	69
70	*Poxviridae*	Pigeonpox virus	GCF_000922075.1	70
71	*Poxviridae*	Turkeypox virus	GCF_001431935.1	71

72	*Poxviridae*	Fowlpox virus	GCF_000838605.1	72
73	*Poxviridae*	Penguinpox virus	GCF_000923135.1	70
74	*Poxviridae*	Goatpox virus Pellor	GCF_000840165.1	73
75	*Poxviridae*	Lumpy skin disease virus NI-2490	GCF_000839805.1	74
76	*Poxviridae*	Sheeppox virus	GCF_000840205.1	73
77	*Poxviridae*	Yoka poxvirus	GCF_000892975.1	75
78	*Poxviridae*	Deerpox virus W-848-83	GCF_000861985.1	76
79	*Poxviridae*	Nile crocodilepox virus	GCF_000869065.1	77
80	*Poxviridae*	Myxoma virus	GCF_000843685.1	78
81	*Poxviridae*	Rabbit fibroma virus	GCF_000847965.1	79
82	*Poxviridae*	Molluscum contagiosum virus subtype 1	GCF_000843325.1	80
83	*Poxviridae*	Camelpox virus	GCF_000839105.1	81
84	*Poxviridae*	Cowpox virus	GCF_000839185.1	82
85	*Poxviridae*	Ectromelia virus	GCF_000841905.1	83
86	*Poxviridae*	Monkeypox virus Zaire-96-I-16	GCF_000857045.1	84
87	*Poxviridae*	Raccoonpox virus	GCF_001029045.1	85
88	*Poxviridae*	Taterapox virus	GCF_000869985.1	86
89	*Poxviridae*	Vaccinia virus	GCF_000860085.1	87
90	*Poxviridae*	Variola virus	GCF_000859885.1	88
91	*Poxviridae*	Bovine papular stomatitis virus	GCF_000844045.1	89
92	*Poxviridae*	Orf virus	GCF_000844845.1	89
93	*Poxviridae*	Parapoxvirus red deer/HL953	GCF_000930695.1	90
94	*Poxviridae*	Pseudocowpox virus	GCF_000886295.1	91
95	*Poxviridae*	Swinepox virus	GCF_000839965.1	92
96	*Poxviridae*	Yaba monkey tumor virus	GCF_000845705.1	93
97	*Poxviridae*	Yaba-like disease virus	GCF_000847185.1	94
98	*Poxviridae*	Squirrelpox virus	GCF_000913615.1	95
99	*Poxviridae*	Cotia virus SPAn232	GCF_000894375.1	96

Supplementary Table 2. NCLDV protein clusters that contributed to new entries in Pfam 33.1 and 34.0 release.

pfamA_acc	pfamA_id	Pfam identifier	Original protein cluster(s)
PF19058	DUF5754	PF19058	cluster_153, cluster_727
PF19061	DUF5757	PF19061	cluster_154
PF19062	DUF5758	PF19062	cluster_158
PF19063	DUF5759	PF19063	cluster_167
PF19064	DUF5760	PF19064	cluster_168, cluster_1213
PF19065	DUF5761	PF19065	cluster_170
PF19066	DUF5762	PF19066	cluster_183, cluster_411, cluster_1328
PF19067	DUF5763	PF19067	cluster_186, cluster_358, cluster_851
PF19068	DUF5764	PF19068	cluster_200, cluster_1484
PF19069	DUF5765	PF19069	cluster_201
PF19070	DUF5766	PF19070	cluster_205
PF19071	DUF5767	PF19071	cluster_213, cluster_332, cluster_489, cluster_1678, cluster_2757
PF19072	DUF5768	PF19072	cluster_214
PF19073	DUF5769	PF19073	cluster_225
PF19074	DUF5770	PF19074	cluster_230
PF19075	DUF5771	PF19075	cluster_234
PF19080	DUF5772	PF19080	cluster_235, cluster_462, cluster_2817
PF19082	DUF5773	PF19082	cluster_250
PF19083	DUF5774	PF19083	cluster_252
PF19084	DUF5775	PF19084	cluster_256
PF19161	DUF5843	PF19161	cluster_400
PF19162	DUF5844	PF19162	cluster_401
PF19163	DUF5845	PF19163	cluster_405
PF19164	DUF5846	PF19164	cluster_431
PF19165	DUF5847	PF19165	cluster_432, cluster_2662, cluster_2663
PF19166	DUF5848	PF19166	cluster_434, cluster_828, cluster_1584, cluster_1990, cluster_1990, cluster_2110, cluster_2439, cluster_2439, cluster_3144, cluster_3207
PF19167	DUF5849	PF19167	cluster_436, cluster_1137, cluster_2128
PF19168	DUF5850	PF19168	cluster_453, cluster_520, cluster_1937
PF19169	DUF5851	PF19169	cluster_812
PF19170	DUF5852	PF19170	cluster_1010
PF19171	DUF5853	PF19171	cluster_979
PF19172	DUF5854	PF19172	cluster_488
PF19173	DUF5855	PF19173	cluster_502
PF19174	DUF5856	PF19174	cluster_515
PF19175	DUF5857	PF19175	cluster_542
PF19176	DUF5858	PF19176	cluster_544
PF19177	DUF5859	PF19177	cluster_591
PF19178	DUF5860	PF19178	cluster_693, cluster_2194, cluster_2195
PF19179	DUF5861	PF19179	cluster_708, cluster_3167
PF19180	DUF5862	PF19180	cluster_718, cluster_2496, cluster_2917
PF19181	DUF5863	PF19181	cluster_575
PF19182	DUF5864	PF19182	cluster_547
PF19183	DUF5865	PF19183	cluster_550, cluster_2104
PF19184	DUF5866	PF19184	cluster_841
PF19185	DUF5867	PF19185	cluster_719
PF19186	DUF5868	PF19186	cluster_685
PF19194	DUF5869	PF19194	cluster_543
PF19195	DUF5870	PF19195	cluster_870
PF19196	DUF5871	PF19196	cluster_930
PF19197	DUF5872	PF19197	cluster_732
PF19201	DUF5873	PF19201	cluster_538
PF19202	DUF5874	PF19202	cluster_375, cluster_713
PF19203	DUF5875	PF19203	cluster_522
PF19204	DUF5876	PF19204	cluster_519
PF19205	DUF5877	PF19205	cluster_516
PF19206	DUF5878	PF19206	cluster_437
PF19207	DUF5879	PF19207	cluster_696
PF19208	DUF5880	PF19208	cluster_564
PF19228	DUF5881	PF19228	cluster_555
PF19229	DUF5882	PF19229	cluster_487
PF19230	DUF5883	PF19230	cluster_529
PF19231	DUF5884	PF19231	cluster_438
PF19232	DUF5885	PF19232	cluster_1097, cluster_2131
PF19233	DUF5886	PF19233	cluster_1077
PF19234	DUF5887	PF19234	cluster_1000
PF19235	DUF5888	PF19235	cluster_535
PF19237	DUF5889	PF19237	cluster_1080 (was listed twice ?)
PF19239	GIY_YIG_dom	PF19239	cluster_858
PF19240	DUF5890	PF19240	cluster_222, cluster_1868
PF19241	DUF5891	PF19241	cluster_271
PF19242	Zn_finger_prot	PF19242	cluster_368
PF19243	DUF5892	PF19243	cluster_792
PF19244	Poly_A_pol_ca	PF19244	cluster_801
PF19245	DUF5893	PF19245	cluster_804, cluster_1912
PF19246	DUF5894	PF19246	cluster_813
PF19247	DUF5895	PF19247	cluster_818
PF19248	DUF5896	PF19248	cluster_823, cluster_3054
PF19249	DUF5897	PF19249	cluster_554, cluster_828
PF19250	DUF5898	PF19250	cluster_831
PF19251	DUF5899	PF19251	cluster_291, cluster_847, cluster_1210, cluster_1465
PF19253	DUF5900	PF19253	cluster_871, cluster_1177, cluster_2155
PF19254	DUF5901	PF19254	cluster_952
PF19255	DUF5902	PF19255	cluster_185, cluster_417
PF19260	DUF5903	PF19260	cluster_308
PF19261	DUF5904	PF19261	cluster_390
PF19262	DUF5905	PF19262	cluster_395
PF19263	DUF5906	PF19263	cluster_1170
PF19264	DUF5907	PF19264	cluster_136

Supplementary Table S3. ORFs of new NCLDVs and their blastp hits to identify protein cluster membership

Species name	Annotated ORFs per genome	Genes receiving a significant BLASTp hit (hit above HSSP curve[97] threshold)	Percentage of the ORFs receiving a blast hit	Number of protein clusters	Number of ORFs hitting singletons
Acanthamoeba castellanii medusavirus	890	220	0.25	88	30
Faustovirus	496	99	0.20	59	18
Tupanvirus deep ocean	1276	726	0.57	463	69
Yaravirus brasiliensis	74	5	0.07	4	0

Supplementary Table S4. The 26 widest distributed protein clusters among NCLDVs.

Order number for the widest distributed protein clusters	Cluster identifier in the dataset*	Pfam**	Pfam annotations	Corresponding protein families in Yutin et. al. 2009 Virol. J. 6:223***	Description
1	cluster_11	PF04947, PF08792, PF17026	A2L zinc ribbon domain, Poxvirus Late Transcription Factor VLTF3 like, Putative ribonucleoprotein zinc-finger pf C4 type	NCVOG0262	Poxvirus Late Transcription Factor VLTF3 like
2	cluster_9	PF04665, PF13401	AAA domain, Poxvirus A32 protein	NCVOG0079, NCVOG0249	packaging ATPase, putative ATPase
3	cluster_3	PF00176, PF00270, PF00271, PF04851, PF05203, PF05204, PF08148, PF08469, PF10544, PF12011, PF13639, PF13920, PF14634, PF14890, PF16124	DEAD/DEAH box helicase, DSHCT (NUC185) domain, Domain of unknown function (DUF3503), Helicase conserved C-terminal domain, Hom_end-associated Hint, Homing endonuclease, Intein splicing domain, Nucleoside triphosphatase I C-terminal, RecQ zinc-binding, Ring finger domain, SNF2 family N-terminal domain, T5orf172 domain, Type III restriction enzyme, res subunit, Zinc finger, C3HC4 type (RING finger), zinc-RING finger domain	NCVOG0025, NCVOG0027, NCVOG0029, NCVOG0030, NCVOG0031, NCVOG0032, NCVOG0076, NCVOG0199, NCVOG0214, NCVOG0267, NCVOG0330, NCVOG1100, NCVOG5431	DEAD-like helicases superfamily, DEAD/SNF2-like helicase, DNA or RNA helicases of superfamily II, NTPase/helicase, RING-finger-containing E3 ubiquitin ligase, RNA-helicase DExH-NPH-II, superfamily II helicase, uncharacterized protein, unclassified DEAD/SNF2-like helicases
4	cluster_12	PF00136, PF02037, PF03104, PF08408, PF08452, PF14890	DNA polymerase family B, DNA polymerase family B exonuclease domain, N-terminal, DNA polymerase family B viral insert, DNA polymerase family B, exonuclease domain, Intein splicing domain, SAP domain	NCVOG0038	DNA polymerase elongation subunit family B
5	cluster_4	PF00069, PF00134, PF05445, PF07714, PF08793, PF13458	2-cysteine adaptor domain, Cyclin, N-terminal domain, Periplasmic binding protein, Poxvirus serine/threonine protein kinase, Protein kinase domain, Protein tyrosine kinase	NCVOG0282, NCVOG0284, NCVOG0288, NCVOG0295, NCVOG0297, NCVOG0301, NCVOG0302, NCVOG0304, NCVOG0305, NCVOG0424, NCVOG1356, NCVOG4292	2-cysteine adaptor domain (pfam08793). The virus-specific domain is found fused to OTU/A20-like peptidases and S/T protein kinases., F10-like kinase, Serine/Threonine or Tyrosine protein kinase (tandem-duplicated in Mimi- and Marseilleviridae), Serine/Threonine protein kinase, Serine/Threonine protein kinases, serine/threonine protein kinase, uncharacterized protein
6	cluster_13	PF04777, PF04805, PF10403	E10-like protein conserved region, Erv1 / Alr family, Rad4 beta-hairpin domain 1	NCVOG0052, NCVOG4692	ERV/alR sulphydryl oxidase-like protein, disulfide (thiol) oxidoreductase, Erv1 / Alr family
7	cluster_15	PF04848, PF09159	Mitochondrial resolvase Ydc2 / RNA splicing MRS1, Poxvirus A22 protein	NCVOG0278, NCVOG4573	RuvC, Holliday junction resolvases (HJRs); Poxvirus A22 family, uncharacterized protein
8	cluster_21	PF00268, PF00462	Glutaredoxin, Ribonucleotide reductase, small chain	NCVOG0276, NCVOG0277, NCVOG0317	Ribonucleotide reductase small subunit, Thioredoxin, ribonucleoside diphosphate reductase, beta subunit
9	cluster_22	PF00692	dUTPase	NCVOG1068	dUTPase
10	cluster_24	PF00035, PF00636, PF02295, PF14622	Adenosine deaminase z-alpha domain, Double-stranded RNA binding motif, Ribonuclease III domain, Ribonuclease-III-like	NCVOG0039, NCVOG1354	Ribonuclease III, double-stranded RNA binding protein (cd00048; pfam00035)
11	cluster_30	PF00317, PF02867, PF03477, PF14890	ATP cone domain, Intein splicing domain, Ribonucleotide reductase, all-alpha domain, Ribonucleotide reductase, barrel domain	NCVOG1353	ribonucleoside diphosphate reductase, alpha subunit
12	cluster_23	PF01712, PF02223, PF04275, PF13238	AAA domain, Deoxynucleoside kinase, Phosphomevalonate kinase, Thymidylate kinase	NCVOG0320, NCVOG1067, NCVOG4130, NCVOG4200	Thymidylate kinase, deoxynucleoside kinase, deoxynucleotide monophosphate kinase (dNMP) kinase, putative deoxynucleoside kinase
13	cluster_28	PF00782	Dual specificity phosphatase, catalytic domain	NCVOG0040	Dual specificity phosphatases (DSP); Ser/Thr and Tyr protein phosphatases
14	cluster_7	PF04451, PF16903	Large eukaryotic DNA virus major capsid protein, Major capsid protein N-terminus	NCVOG0022	NCLDV major capsid protein
15	cluster_1	PF00023, PF00646, PF03158, PF09372, PF12796, PF12937, PF13606, PF13637, PF13639, PF13857	Ankyrin repeat, Ankyrin repeats (3 copies), Ankyrin repeats (many copies), F-box domain, F-box-like, Multigene family 530 protein, PRANC domain, Ring finger domain	NCVOG0021, NCVOG0057, NCVOG0170, NCVOG0171, NCVOG0172, NCVOG0173, NCVOG0174, NCVOG0175, NCVOG0176, NCVOG0177, NCVOG0178, NCVOG0179, NCVOG0244, NCVOG0330, NCVOG0446, NCVOG0447, NCVOG0448, NCVOG0449, NCVOG0450, NCVOG0451, NCVOG0496, NCVOG0497, NCVOG0528, NCVOG0684, NCVOG0699, NCVOG0710, NCVOG0822, NCVOG0825, NCVOG1013, NCVOG1157, NCVOG1303, NCVOG1407, NCVOG2058, NCVOG2443, NCVOG2734, NCVOG2775, NCVOG2860, NCVOG4001, NCVOG4104, NCVOG4108, NCVOG4109, NCVOG4111, NCVOG4112, NCVOG4115, NCVOG4116, NCVOG4118, NCVOG4121, NCVOG4122, NCVOG4123, NCVOG4344, NCVOG4371, NCVOG4372, NCVOG4382, NCVOG4404, NCVOG4412, NCVOG4413, NCVOG4414, NCVOG4416, NCVOG4418, NCVOG4419, NCVOG4420, NCVOG4421, NCVOG4422, NCVOG4423, NCVOG4430, NCVOG4441, NCVOG4444, NCVOG4445, NCVOG4459, NCVOG4467, NCVOG4488, NCVOG4524, NCVOG4579, NCVOG4590, NCVOG4624, NCVOG4629, NCVOG4676, NCVOG4803, NCVOG4844, NCVOG4871, NCVOG5240, NCVOG5256, NCVOG5265, NCVOG5270, NCVOG5272, NCVOG5273, NCVOG5275, NCVOG5278, NCVOG5280, NCVOG5297, NCVOG5299, NCVOG5301, NCVOG5309, NCVOG5317, NCVOG5320, NCVOG5323, NCVOG5324, NCVOG5338, NCVOG5340, NCVOG5341, NCVOG5346, NCVOG5375, NCVOG5381, NCVOG5385, NCVOG5386, NCVOG5388, NCVOG5389, NCVOG5390, NCVOG5392, NCVOG5393, NCVOG5397, NCVOG5401, NCVOG5402, NCVOG5403, NCVOG5404	F-box domain protein, Fbox-containing protein, RING-finger-containing E3 ubiquitin ligase, ankyrin repeat protein, collagen triple helix repeat containing protein, hypothetical protein, similar to ankyrin, uncharacterized protein
16	cluster_45	PF01096, PF07500	Transcription factor S-II (TFIIS), Transcription factor S-II (TFIIS), central domain	NCVOG0272	Transcription factor S-II (TFIIS)
17	cluster_58	PF00204, PF00521, PF01751, PF02518, PF16898	C-terminal associated domain of TOPRIM, DNA gyrase B, DNA gyrase/topoisomerase IV, subunit A, Histidine kinase-, DNA gyrase B-, and HSP90-like ATPase, Toprim domain	NCVOG0037	DNA topoisomerase II
18	cluster_50	PF00623, PF04983, PF04990, PF04997, PF04998, PF05000, PF14890	Intein splicing domain, RNA polymerase Rpb1, domain 1, RNA polymerase Rpb1, domain 2, RNA polymerase Rpb1, domain 3, RNA polymerase Rpb1, domain 4, RNA polymerase Rpb1, domain 5, RNA polymerase Rpb1, domain 7	NCVOG0274	DNA-directed RNA polymerase subunit alpha
19	cluster_55	PF00562, PF04560, PF04561, PF04563, PF04565, PF04566, PF04567	RNA polymerase Rpb2, domain 2, RNA polymerase Rpb2, domain 3, RNA polymerase Rpb2, domain 4, RNA polymerase Rpb2, domain 5, RNA polymerase Rpb2, domain 6, RNA polymerase Rpb2, domain 7, RNA polymerase beta subunit	NCVOG0271	DNA-directed RNA polymerase subunit beta
20	cluster_37	PF00752, PF00867, PF03159	XPG I-region, XPG N-terminal domain, XRN 5'-3' exonuclease N-terminus	NCVOG1045, NCVOG1060	FLAP-like endonuclease XPG, XRN 5'-3' exonuclease
21	cluster_57	PF01734	Patatin-like phospholipase	NCVOG0245	Patatin phospholipase
22	cluster_89	PF08706, PF08707	D5 N terminal like, Primase C terminal 2 (PriCT-2)	NCVOG0023	D5-like helicase-primase
23	cluster_146	PF00075, PF01693	Caulimovirus viroplasmin, RNase H	NCVOG1352, NCVOG4654	RNAseH1 eukaryote like-protein, ribonuclease H
24	cluster_145	PF02689, PF05970, PF13538, PF13604	AAA domain, Helicase, PIF1-like helicase, UvrD-like helicase C-terminal domain	NCVOG0248	Pif1 helicase
25	cluster_94	PF00069, PF00211, PF07714, PF12849, PF13458	Adenylate and Guanylate cyclase catalytic domain, PBP superfamily domain, Periplasmic binding protein, Protein kinase domain, Protein tyrosine kinase	NCVOG0295, NCVOG0305, NCVOG0307	F10-like kinase, Serine/Threonine or Tyrosine protein kinase (tandem-duplicated in Mimi- and Marseilleviridae), tyrosine protein kinase
26	cluster_179	PF00240	Ubiquitin family	NCVOG0333	Ubiquitin

* Identifier of the protein cluster in this study

**Pfam annotations are based on Interproscan[10] annotations with default parameters. All the pfam annotations for protein family members are shown.

***The protein family identifiers based on Yutin et al 2009[98]. Protein familes are given only for those proteins that were included to the study Yutin et al. 2009.

Supplementary Table S5. Protein included in the phylogeneies following contaminant screen for eukaryote protein and their scaffolds that form individual branches in the phylogenetic trees for cluster_11, cluster_9, cluster_13 and cluster_15.

The order number for the widest distributed protein clusters among NCLDVs	Supplementary Figure	Cluster identifier in the dataset	Protein id	Species name	Scaffold	Size (bp)	Number of ORFs in the same scaffold	Proportion of eukaryotic proteins (eukaryotic hits/ (cellular + viral hits))	Eukaryotic hits	Bacterial hits	Archaeal hits	Viral hits	No hit
1	S3	Cluster_11	CBN80403.1	*Ectocarpus siliculosus*	FN649741.1	8064047	1840	61.56	1134	50	0	340	316
1	S3	Cluster_11	GAQ85075.1	*Klebsormidium nitens*	DF237168.1	137706	201	80.6	162	0	0	6	33
1	S3	Cluster_11	GAQ90999.1	*Klebsormidium nitens*	DF237661.1	47014	36	83.33	30	0	0	2	4
1	S3	Cluster_11	GAQ92093.1	*Klebsormidium nitens*	DF237869.1	29316	24	66.67	16	0	0	0	8
1	S3	Cluster_11	GAQ93349.1	*Klebsormidium nitens*	DF238410.1	5700	3	66.67	2	0	0	0	1
1	S3	Cluster_11	KXS14114.1	*Gonapodya prolifera* JEL478	KQ965771.1	357412	152	65.79	100	4	0	1	47
1	S3	Cluster_11	XP_004205627.1	*Hydra vulgaris*	NW_004166914.1	395687	22	27.27	6	4	0	8	4
1	S3	Cluster_11	XP_005836760.1	*Guillardia theta* CCMP2712	NW_005434657.1	1003429	562	66.08	374	18	2	2	166
2	S4	Cluster_9	CBN80334.1	*Ectocarpus siliculosus*	FN649741.1	8064047	1840	61.56	1134	50	0	340	316
2	S4	Cluster_9	ETO03050.1	*Reticulomyxa filosa*	ASPP01034267.1	9090	5	80	4	0	0	0	1
2	S4	Cluster_9	GAQ85072.1	*Klebsormidium nitens*	DF237168.1	137706	201	80.6	162	0	0	6	33
2	S4	Cluster_9	GAQ90578.1	*Klebsormidium nitens*	DF237607.1	50556	15	86.67	13	0	0	0	2
2	S4	Cluster_9	GAQ91460.1	*Klebsormidium nitens*	DF237738.1	32734	12	75	9	0	0	0	3
2	S4	Cluster_9	GAQ91699.1	*Klebsormidium nitens*	DF237785.1	30433	11	54.55	6	0	0	0	5
2	S4	Cluster_9	GAQ91766.1	*Klebsormidium nitens*	DF237797.1	28847	8	37.5	3	0	0	0	5
2	S4	Cluster_9	XP_014157608.1	*Sphaeroforma arctica* JP610	NW_014040339.1	112087	17	70.59	12	0	0	1	4
6	S6	Cluster_13	XP_005702698.1	*Galdieria sulphuraria*	NW_005178373.1	10166	7	14.29	1	1	0	2	3
7	S7	Cluster_15	CBN80409.1	*Ectocarpus siliculosus*	FN649741.1	8064047	1840	61.56	1134	50	0	340	316
7	S7	Cluster_15	GAQ85074.1	Klebsormidium nitens	DF237168.1	137706	201	80.6	162	0	0	6	33
7	S7	Cluster_15	GAQ87941.1	Klebsormidium nitens	DF237338.1	89163	32	78.12	25	0	0	1	6
7	S7	Cluster_15	GAQ89989.1	Klebsormidium nitens	DF237535.1	55443	20	60	12	0	0	0	8
7	S7	Cluster_15	GAQ90998.1	Klebsormidium nitens	DF237661.1	47014	36	83.33	30	0	0	2	4
7	S7	Cluster_15	GAQ92094.1	Klebsormidium nitens	DF237869.1	29316	24	66.67	16	0	0	0	8

Supplementary Table 6A. Most widely shared annotated protein clusters that are unique to NCLDVs

Number of NCLDV families that have the cluster	Number of NCLDV species that have a protein family member[a]	Protein cluster	Pfam id [101]	Pfam annotation (and function)
4	47	cluster_16	PF02442	Lipid membrane protein of large eukaryotic DNA viruses
2	43	cluster_53	PF03292	Poxvirus P4B major core protein
2	42	cluster_47	PF03003	Pox virus entry-fusion-complex G9/A16
1	39	cluster_64	PF03410	Metallopeptidase from vaccinia pox
1	39	cluster_65	PF03293	Poxvirus DNA-directed RNA polymerase, 18 kD subunit
1	39	cluster_66	PF03296	Poxvirus poly(A) polymerase nucleotidyltransferase domain
1	39	cluster_70	PF05323	Poxvirus A21 Protein (Envelope protein part of the entry-fusion complex responsible for the virus membrane fusion with host cell membrane during virus entry) [99]
1	39	cluster_73	PF04441	Poxvirus early transcription factor (VETF), large subunit
1	39	cluster_74	PF03340	Poxvirus rifampicin resistance protein, Essential for replication[100]
1	39	cluster_75	PF04584	Poxvirus A28 family; (Virus penetration into host cytoplasm)[101]

[a] Annotated using interproscan[10] against Pfam (v29.0) database[102]

Supplementary Table 6B. Proportion of annotated NCLDV specific protein clusters in different NCLDV fa

Virus family / genus	Annotated NCLDV specific protein clusters / Total number of NCLDV specific protein clusters in a virus family / genus (%)
Ascoviridae	10.91
Asfarviridae	33.33
Iridoviridae	6.54
Marseilleviridae	7.17
Mimiviridae	8.06
Mollivirus	4.35
Pandoravirus	5.01
Phycodnaviridae	3.88
Pithovirus	21.74
Poxviridae	32.47

Supplementary Table S7. Protein clusters that are found only among insect-infecting

Protein cluster	Virus family or cellular life domain	Pfam id	Pfam annotation	Protein family based on Yutin et al. 2014.[98]	Protein family annotations based on Yutin et al. 2014.[98]
cluster_164	Ascoviridae, Poxviridae	-, PF12678, PF13639, PF13920	-, RING-H2 zinc finger domain, Ring finger domain, Zinc finger, C3HC4 type (RING finger)	-, NCVOG0330	-, RING-finger-containing E3 ubiquitin ligase
cluster_197	Ascoviridae, Iridoviridae, Poxviridae	-, PF00413	-, Matrixin	-, NCVOG1120	-, metallopeptidase WLM
cluster_229	Ascoviridae, Baculoviridae, Poxviridae	-, PF13930	-, DNA/RNA non-specific endonuclease	-, NCVOG1417	-, uncharacterized protein
cluster_233	Iridoviridae, Poxviridae	PF10544, PF10553	MSV199 domain, T5orf172 domain	-, NCVOG1361	-, T5orf172 domain
cluster_298	Ascoviridae, Baculoviridae, Eukaryota, Iridoviridae, Poxviridae	PF00653, PF13920	Inhibitor of Apoptosis domain, Zinc finger, C3HC4 type (RING finger)	-, NCVOG0009, NCVOG4003, NCVOG5020	-, inhibitor of apoptosis protein, uncharacterized protein
cluster_306	Ascoviridae, Iridoviridae	-	-	-, NCVOG1086	-, uncharacterized protein
cluster_355	Ascoviridae, Iridoviridae	-	-	-, NCVOG4936	-, uncharacterized protein
cluster_368	Ascoviridae, Iridoviridae	-	-	-, NCVOG5007	-, uncharacterized protein
cluster_369	Ascoviridae, Iridoviridae	-	-	-, NCVOG4921	-, uncharacterized protein
cluster_388	Ascoviridae, Iridoviridae	-	-	-, NCVOG1164	-, A1L transcription factor/late transcription factor VLTF-2
cluster_389	Ascoviridae, Iridoviridae	-	-	-, NCVOG0161	-, uncharacterized protein
cluster_416	Baculoviridae, Poxviridae	-	-	-, NCVOG0926	-, uncharacterized protein
cluster_423	Baculoviridae, Poxviridae	-, PF02331	-, Apoptosis preventing protein	-, NCVOG5229	-, uncharacterized protein
cluster_430	Baculoviridae, Poxviridae	-	-	-, NCVOG0135, NCVOG5196	-, uncharacterized protein
cluster_561	Iridoviridae, Poxviridae	-, PF04973	-, Nicotinamide mononucleotide transporter	-, NCVOG4002	-, uncharacterized protein
cluster_575	Ascoviridae, Baculoviridae, Iridoviridae	-	-	-, NCVOG4380	-, uncharacterized protein
cluster_587	Ascoviridae, Baculoviridae	-, PF07282	-, Putative transposase DNA-binding domain	NCVOG0327	uncharacterized protein
cluster_661	Ascoviridae, Baculoviridae	-, PF02498	-, BRO family, N-terminal domain	NCVOG0010, NCVOG5016	BRO-like protein, uncharacterized protein
cluster_665	Baculoviridae, Iridoviridae, Poxviridae	-	-	-, NCVOG4990	-, uncharacterized protein
cluster_734	Iridoviridae, Poxviridae	PF13489, PF13649, PF13847	Methyltransferase domain	-, NCVOG1191	-, SAM dependent methyltransferase
cluster_780	Baculoviridae, Poxviridae	-	-	-, NCVOG0106	-, uncharacterized protein
cluster_783	Baculoviridae, Eukaryota, Poxviridae	-, PF03067	-, Lytic polysaccharide mono-oxygenase, cellulose-degrading	-, NCVOG4996	-, fusolin
cluster_818	Ascoviridae, Iridoviridae	-	-	-, NCVOG1085	-, uncharacterized protein
cluster_822	Ascoviridae, Iridoviridae	-	-	-, NCVOG1052	-, uncharacterized protein
cluster_993	Baculoviridae, Poxviridae	PF08087	Conotoxin O-superfamily	-, NCVOG4952	-, conotoxin-like protein
cluster_1000	Baculoviridae, Poxviridae	-	-	-, NCVOG5224	-, uncharacterized protein
cluster_1116	Ascoviridae, Baculoviridae, Eukaryota, Poxviridae	PF13920	Zinc finger, C3HC4 type (RING finger)	-, NCVOG4379, NCVOG4944, NCVOG4947	-, uncharacterized protein
cluster_1242	Baculoviridae, Poxviridae	-	-	-	-
cluster_1423	Iridoviridae, Poxviridae	-	-	-	-
cluster_1451	Ascoviridae, Polydnaviridae	-	-	NCVOG1054	uncharacterized protein
cluster_1452	Ascoviridae, Baculoviridae, Iridoviridae	-	-	-, NCVOG0119	-, uncharacterized protein
cluster_1561	Ascoviridae, Iridoviridae	-, PF08707, PF09250	-, Bifunctional DNA primase/polymerase, N-terminal, Primase C terminal 2 (PriCT-2)	-	-
cluster_1849	Bacteria, Baculoviridae, Iridoviridae	PF10544	T5orf172 domain	-, NCVOG1361	-, T5orf172 domain
cluster_1851	Ascoviridae, Iridoviridae	-, PF14608	-, RNA-binding, Nab2-type zinc finger	-	-
cluster_1887	Ascoviridae, Baculoviridae	-	-	NCVOG0160	uncharacterized protein
cluster_2302	Ascoviridae, Iridoviridae	-	-	-	-
cluster_2309	Baculoviridae, Poxviridae	-, PF13639, PF13920	-, Ring finger domain, Zinc finger, C3HC4 type (RING finger)	-	-
cluster_2383	Ascoviridae, Baculoviridae	-, PF00656	-, Caspase domain	NCVOG1053	caspase-like protein
cluster_2808	Baculoviridae, Poxviridae	PF09414	RNA ligase	NCVOG5235	uncharacterized protein
cluster_2811	Ascoviridae, Poxviridae	PF10488	Phosphatase-1 catalytic subunit binding region	NCVOG0965	protein phosphatase 1, regulatory subunit 15A
cluster_2820	Ascoviridae, Baculoviridae, Iridoviridae	PF09511	RNA ligase	-, NCVOG1088	-, RNA ligase (conserved in irido-, asfa-, asco- and Marseille viruses)
cluster_2963	Ascoviridae, Baculoviridae	-, PF10544	-, T5orf172 domain	NCVOG1361	T5orf172 domain
cluster_2978	Ascoviridae, Baculoviridae	-	-	NCVOG5016	uncharacterized protein
cluster_2980	Ascoviridae, Baculoviridae	-	-	NCVOG0959	uncharacterized protein
cluster_2981	Ascoviridae, Baculoviridae, Eukaryota, Poxviridae	PF13164	Protein of unknown function (DUF4002)	NCVOG0377	uncharacterized protein
cluster_2992	Ascoviridae, Baculoviridae	PF13639	Ring finger domain	NCVOG0330	RING-finger-containing E3 ubiquitin ligase
cluster_3007	Iridoviridae, Poxviridae	-	-	NCVOG4928	uncharacterized protein
unclassified YP_001110887.1	Ascoviridae, Baculoviridae	-	-	NCVOG0328	uncharacterized protein
unclassified YP_001110896.1	Ascoviridae, Baculoviridae	-	-	-	-
unclassified YP_001110941.1	Ascoviridae, Baculoviridae	-	-	-	-
unclassified YP_001110943.1	Ascoviridae, Baculoviridae	-	-	NCVOG0010	BRO-like protein
unclassified YP_001110950.1	Ascoviridae, Baculoviridae	PF02498	BRO family, N-terminal domain	NCVOG0010	BRO-like protein
unclassified YP_001110964.1	Ascoviridae, Baculoviridae	-	-	NCVOG0010	BRO-like protein
unclassified YP_001110976.1	Ascoviridae, Baculoviridae	-	-	-	-
unclassified YP_001110977.1	Ascoviridae, Baculoviridae	-	-	-	-
unclassified YP_001111014.1	Ascoviridae, Baculoviridae	PF10544	T5orf172 domain	NCVOG1361	T5orf172 domain
unclassified YP_008003527.1	Baculoviridae, Poxviridae	-	-	-	-
unclassified YP_008003798.1	Baculoviridae, Poxviridae	-	-	NCVOG5235	uncharacterized protein
unclassified YP_008003801.1	Baculoviridae, Poxviridae	PF13930	DNA/RNA non-specific endonuclease	NCVOG1417	uncharacterized protein
unclassified YP_008003811.1	Baculoviridae, Poxviridae	-	-	-	-
unclassified YP_008004018.1	Baculoviridae, Poxviridae	-	-	-	-
unclassified YP_762386.1	Ascoviridae, Baculoviridae	-	-	-	-
unclassified YP_762426.1	Ascoviridae, Baculoviridae	-	-	-	-
unclassified YP_762435.1	Ascoviridae, Baculoviridae	PF02498	BRO family, N-terminal domain	NCVOG0010	BRO-like protein
unclassified YP_762476.1	Ascoviridae, Baculoviridae	PF13164	Protein of unknown function (DUF4002)	-	-
unclassified YP_803313.1	Ascoviridae, Baculoviridae	-	-	-	-
unclassified YP_803353.1	Ascoviridae, Baculoviridae	-	-	-	-

Supplementary Table S8. Protein clusters that are found only from vertebrate-infecting viruses (present at least in two virus families).

Protein cluster	Virus family or cellular life domain	Pfam id	Pfam annotation	Protein family based on Yutin et al. 2014.	Protein family annotations based on Yutin et al. 2014.
cluster_486	Herpesviridae, Poxviridae	PF12906	RING-variant domain	-, NCVOG1394	-, uncharacterized protein
cluster_675	Iridoviridae, Poxviridae	PF00341	PDGF/VEGF domain	-, NCVOG0423	-, vascular endothelial growth factor-like protein
cluster_758	Herpesviridae, Poxviridae	-	-	-, NCVOG0885	-, uncharacterized protein
cluster_1573	Eukaryota, Herpesviridae, Poxviridae	-, PF00059	-, Lectin C-type domain	-	-
cluster_1710	Herpesviridae, Poxviridae	-	-	-, NCVOG0309	-, semaphorin-like protein
cluster_1844	Iridoviridae, Poxviridae	-	-	-	-
cluster_1865	Iridoviridae, Poxviridae	PF01027	Inhibitor of apoptosis-promoting Bax1	-	-
cluster_2914	Alloherpesviridae, Iridoviridae, Poxviridae	PF13168	Poxvirus B22R protein C-terminal	-	-
cluster_3081	Iridoviridae, Poxviridae	-	-	-, NCVOG1196	-, uncharacterized protein
unclassified YP_009162534.1	Alloherpesviridae, Poxviridae	PF13168	Poxvirus B22R protein C-terminal	-	-
unclassified YP_784345.1	Alloherpesviridae, Poxviridae	-	-	-	-

Supplementary Table S9. Protein clusters that are shared with phages.

Protein cluster	Virus family or cellular life domain	Pfam id	Pfam annotation	Protein family based on Yutin et al. 2014.	Protein family annotations based on Yutin et al. 2014.
cluster_2	Ascoviridae, Bacteria, Baculoviridae, Eukaryota, Iridoviridae, Mimiviridae, Poxviridae, Siphoviridae	PF01541, PF02498, PF02796, PF03288, PF04218, PF04383, PF04480, PF10544, PF10553, PF12299, PF13639	BRO family, N-terminal domain, CENP-B N-terminal DNA-binding domain, GIY-YIG catalytic domain, Helix-turn-helix domain of resolvase, KilA-N domain, MSV199 domain, Poxvirus D5 protein-like, Protein of unknown function (DUF3627), Protein of unknown function (DUF559), Ring finger domain, T5orf172 domain	NCVOG0010, NCVOG0230, NCVOG1360, NCVOG1361, NCVOG1364, NCVOG1386, NCVOG1424, NCVOG5210, NCVOG5406, NCVOG5420, NCVOG5421, NCVOG5424, NCVOG5426, NCVOG5427, NCVOG5428, NCVOG5429, NCVOG5430, NCVOG5433, NCVOG5435, NCVOG5436	ALI motif family protein, ALI motif gene family protein, BRO-N domain protein, BRO-like protein, KilA N-domain protein, KilA domain (pfam04383), KilA-N domain protein, MTG motif gene family protein, T5orf172 domain, putative RING finger host range protein, uncharacterized domain DUF3627; found downstream KilA, BRO, and MSV199 domains. Also is found in some baculoviruses (gi 165969059, 18138388), uncharacterized protein, zinc finger-like protein
cluster_3	Archaea, Ascoviridae, Asfarviridae, Bacteria, Eukaryota, Iridoviridae, Marseilleviridae, Mimiviridae, Mollivirus, Pandoravirus, Phycodnaviridae, Pithovirus, Poxviridae, Siphoviridae	PF00176, PF00270, PF00271, PF04851, PF05203, PF05204, PF08148, PF08469, PF10544, PF12011, PF13639, PF13920, PF14634, PF14890, PF16124	DEAD/DEAH box helicase, DSHCT (NUC185) domain, Domain of unknown function (DUF3503), Helicase conserved C-terminal domain, Hom_end-associated Hint, Homing endonuclease, Intein splicing domain, Nucleoside triphosphatase I C-terminal, RecQ zinc-binding, Ring finger domain, SNF2 family N-terminal domain, T5orf172 domain, Type III restriction enzyme, res subunit, Zinc finger, C3HC4 type (RING finger), zinc-RING finger domain	NCVOG0025, NCVOG0027, NCVOG0029, NCVOG0030, NCVOG0031, NCVOG0032, NCVOG0076, NCVOG0199, NCVOG0214, NCVOG0267, NCVOG0330, NCVOG1100, NCVOG5431	DEAD-like helicases superfamily, DEAD/SNF2-like helicase, DNA or RNA helicases of superfamily II, NTPase/helicase, RING-finger-containing E3 ubiquitin ligase, RNA-helicase DExH-NPH-II, superfamily II helicase, uncharacterized protein, unclassified DEAD/SNF2-like helicases
cluster_19	Archaea, Bacteria, Eukaryota, Iridoviridae, Mimiviridae, Myoviridae, Phycodnaviridae, Poxviridae, Siphoviridae	PF00908, PF01073, PF01370, PF02719, PF04321, PF16363	3-beta hydroxysteroid dehydrogenase/isomerase family, GDP-mannose 4,6 dehydratase, NAD dependent epimerase/dehydratase family, Polysaccharide biosynthesis protein, RmlD substrate binding domain, dTDP-4-dehydrorhamnose 3,5-epimerase	NCVOG1049	short chain dehydrogenase
cluster_23	Ascoviridae, Asfarviridae, Bacteria, Eukaryota, Iridoviridae, Marseilleviridae, Mimiviridae, Myoviridae, Phycodnaviridae, Pithovirus, Poxviridae	PF01712, PF02223, PF04275, PF13238	AAA domain, Deoxynucleoside kinase, Phosphomevalonate kinase, Thymidylate kinase	NCVOG0320, NCVOG1067, NCVOG4130, NCVOG4200	Thymidylate kinase, deoxynucleoside kinase, deoxynucleotide monophosphate kinase (dNMP) kinase, putative deoxynucleoside kinase
cluster_27	Bacteria, Eukaryota, Marseilleviridae, Myoviridae, Pandoravirus, Phycodnaviridae, Siphoviridae, unclassified phages	PF01541, PF07453, PF07460	GIY-YIG catalytic domain, NUMOD1 domain, NUMOD3 motif (2 copies)	NCVOG0062, NCVOG1046	GIY-YIG-like endonuclease, Zn-finger protein
cluster_30	Ascoviridae, Asfarviridae, Bacteria, Eukaryota, Iridoviridae, Marseilleviridae, Mimiviridae, Pandoravirus, Phycodnaviridae, Pithovirus, Podoviridae, Poxviridae, Siphoviridae	PF00317, PF02867, PF03477, PF14890	ATP cone domain, Intein splicing domain, Ribonucleotide reductase, all-alpha domain, Ribonucleotide reductase, barrel domain	NCVOG1353	ribonucleoside diphosphate reductase, alpha subunit
cluster_34	Bacteria, Eukaryota, Mimiviridae, Myoviridae, Phycodnaviridae, Poxviridae	PF01755, PF03213	Glycosyltransferase family 25 (LPS biosynthesis protein), Poxvirus P35 protein	NCVOG0256, NCVOG2945, NCVOG4075	IMV envelope protein p35, glycosyltransferase family protein, uncharacterized protein
cluster_42	Bacteria, Mimiviridae, Myoviridae, Phycodnaviridae	PF13640	2OG-Fe(II) oxygenase superfamily	NCVOG1166, NCVOG4176, NCVOG5075, NCVOG5166, NCVOG5167	2OG-Fe(II) oxygenase family protein, dioxygenase, uncharacterized protein
cluster_46	Bacteria, Eukaryota, Marseilleviridae, Mimiviridae, Myoviridae, Phycodnaviridae, Podoviridae, Siphoviridae	PF07463, PF13392	HNH endonuclease, NUMOD4 motif	NCVOG0072	HNH endonuclease
cluster_127	Bacteria, Myoviridae, Phycodnaviridae	-	-	NCVOG1220	uncharacterized protein
cluster_153	Eukaryota, Iridoviridae, Phycodnaviridae, unclassified phages	-	-	NCVOG1349	uncharacterized protein
cluster_164	Ascoviridae, Bacteria, Eukaryota, Myoviridae, Poxviridae	PF12678, PF13639, PF13920	RING-H2 zinc finger domain, Ring finger domain, Zinc finger, C3HC4 type (RING finger)	NCVOG0330	RING-finger-containing E3 ubiquitin ligase
cluster_176	Bacteria, Eukaryota, Iridoviridae, Myoviridae	-	-	NCVOG0967	uncharacterized protein
cluster_188	Bacteria, Eukaryota, Mimiviridae, Mollivirus, Phycodnaviridae, Siphoviridae	PF00383	Cytidine and deoxycytidylate deaminase zinc-binding region	NCVOG1064	Cytidine and deoxycytidylate deaminase
cluster_210	Asfarviridae, Bacteria, Marseilleviridae, Myoviridae, Phycodnaviridae, Pithovirus, unclassified phages	PF01068, PF14743	ATP dependent DNA ligase domain, DNA ligase OB-like domain	NCVOG0034	-ATP-dependent DNA ligase
cluster_211	Bacteria, Eukaryota, Iridoviridae, Marseilleviridae, Myoviridae	-	-	NCVOG1075	MutH/Vsr/archaeal HJR-like endonuclease
cluster_223	Ascoviridae, Bacteria, Iridoviridae, Marseilleviridae, Myoviridae, Pithovirus, unclassified phages	PF00149	Calcineurin-like phosphoesterase	NCVOG0308	DNA repair exonuclease
cluster_240	Bacteria, Eukaryota, Phycodnaviridae, Pithovirus, Podoviridae, Siphoviridae	PF02511	Thymidylate synthase complementing protein	NCVOG1346	FAD-dependent thymidylate synthase
cluster_244	Ascoviridae, Bacteria, Eukaryota, Iridoviridae, Mimiviridae, Myoviridae, Phycodnaviridae	PF00293	NUDIX domain	NCVOG0236, NCVOG4434, NCVOG4453	Nudix hydrolase (D10 ortholog), putative NUDIX hydrolase
cluster_315	Myoviridae, Phycodnaviridae	-	-	NCVOG2191	uncharacterized protein
cluster_352	Bacteria, Eukaryota, Myoviridae, Phycodnaviridae	PF01041	DegT/DnrJ/EryC1/StrS aminotransferase family	NCVOG4024	uncharacterized protein
cluster_384	Eukaryota, Mimiviridae, Pandoravirus, Phycodnaviridae, Siphoviridae	-	-	NCVOG1046	Zn-finger protein
cluster_429	Bacteria, Eukaryota, Mimiviridae, Myoviridae	-	-	NCVOG2295	uncharacterized protein
cluster_515	Bacteria, Myoviridae, Phycodnaviridae	-	-	NCVOG1239	uncharacterized protein
cluster_730	Bacteria, Phycodnaviridae, Siphoviridae	PF03013	Pyrimidine dimer DNA glycosylase	NCVOG1291	putative endonuclease
cluster_873	Bacteria, Phycodnaviridae, Siphoviridae	PF13385	Concanavalin A-like lectin/glucanases superfamily	-	-
cluster_1037	Myoviridae, Phycodnaviridae	PF01467	Cytidylyltransferase-like	NCVOG4054	uncharacterized protein
cluster_1080	Mimiviridae, Myoviridae	-	-	NCVOG0737, NCVOG4452	-uncharacterized protein
cluster_1086	Bacteria, Eukaryota, Myoviridae, Phycodnaviridae	PF00565	Staphylococcal nuclease homologue	NCVOG1023	uncharacterized protein
cluster_1120	Bacteria, Myoviridae, Phycodnaviridae	PF13759	Putative 2OG-Fe(II) oxygenase	-	-
cluster_1256	Bacteria, Mimiviridae, Myoviridae, Phycodnaviridae	PF02739	5'-3' exonuclease, N-terminal resolvase-like domain	NCVOG2640	putative RNaseH
cluster_1515	Bacteria, Myoviridae, Phycodnaviridae	PF13884	Chaperone of endosialidase	NCVOG4129	uncharacterized protein
cluster_1553	Myoviridae, Phycodnaviridae	-	-	-	-
cluster_1687	Bacteria, Mimiviridae, Myoviridae	PF01503	Phosphoribosyl-ATP pyrophosphohydrolase	NCVOG0640	Nucleoside Triphosphate Pyrophosphohydrolase
cluster_1737	Mimiviridae, Siphoviridae	PF13392	HNH endonuclease	NCVOG2487	Numod4 NHN endonuclease
cluster_1961	Bacteria, Eukaryota, Myoviridae, Phycodnaviridae	PF13884	Chaperone of endosialidase	-	-
cluster_2475	Bacteria, Phycodnaviridae, Siphoviridae	PF13385	Concanavalin A-like lectin/glucanases superfamily	NCVOG0107	uncharacterized protein
cluster_2532	Bacteria, Myoviridae, Phycodnaviridae	PF04519	Polymer-forming cytoskeletal	NCVOG3007	uncharacterized protein
cluster_2587	Phycodnaviridae, Siphoviridae	-	-	-	-
cluster_2910	Bacteria, Phycodnaviridae, Siphoviridae	PF04434, PF07659	Domain of Unknown Function (DUF1599), SWIM zinc finger	NCVOG3095	uncharacterized protein
cluster_3056	Marseilleviridae, Myoviridae	PF13392	HNH endonuclease	NCVOG0072	HNH endonuclease
cluster_3369	Myoviridae, Phycodnaviridae	-	-	-	-
unclassified YP_009172561.1	Myoviridae, Phycodnaviridae	PF16778	Phage tail assembly chaperone protein	-	-
unclassified YP_009172594.1	Phycodnaviridae, Podoviridae	-	-	-	-
unclassified YP_004061598.1	Myoviridae, Phycodnaviridae	-	-	NCVOG5117	uncharacterized protein
unclassified NP_149563.1	Iridoviridae, Podoviridae	PF00644	Poly(ADP-ribose) polymerase catalytic domain	-	-
unclassified YP_009174846.1	Myoviridae, Phycodnaviridae	PF13640	2OG-Fe(II) oxygenase superfamily	-	-
unclassified YP_009174389.1	Phycodnaviridae, Podoviridae	-	-	-	-
unclassified YP_009174463.1	Myoviridae, Phycodnaviridae	PF13884	Chaperone of endosialidase	-	-

Supplementary Table S10. Protein clusters that are shared with virophages.

Protein cluster	Virus family or cellular life domain	Pfam id	Pfam annotation	Protein family based on Yutin et al. 2014.	Protein family annotations based on Yutin et al. 2014.
cluster_72	Bacteria, Poxviridae, virophages	PF03288, PF08706	D5 N-terminal-like, Poxvirus D5 protein-like	NCVOG0023	D5-like helicase-primase
cluster_177	Bacteria, Phycodnaviridae, virophages	-	-	NCVOG5074, NCVOG5083, NCVOG5121, NCVOG5165	uncharacterized protein
cluster_674	Phycodnaviridae, virophages	PF01541	GIY-YIG catalytic domain	NCVOG4163, NCVOG4962	GIY-YIG superfamily protein, uncharacterized protein
cluster_732	Phycodnaviridae, virophages	-	-	NCVOG1231	uncharacterized protein
cluster_968	Eukaryota, Phycodnaviridae, virophages	PF06598, PF13884	Chaperone of endosialidase, Chlorovirus glycoprotein repeat	NCVOG0018, NCVOG1226	Chlorovirus glycoprotein repeat, glycoprotein repeat protein
cluster_1729	Mimiviridae, virophages	-	-	NCVOG0680	uncharacterized protein
cluster_3006	Iridoviridae, virophages	-	-	-	-
cluster_3390	Phycodnaviridae, virophages	PF13920	Zinc finger, C3HC4 type (RING finger)	-	-
unclassified YP_004894054.1	Mimiviridae, virophages	-	-	-	-

References for Supplementary Data

1. Li L, Stoeckert J. Jr, Roos DS. OrthoMCL: Identification of ortholog groups for eukaryotic genomes. *Genome Res* 2003; *13: 2178-2189.*

2. Sievers F, Wilm A, Dineen DG, Gibson TJ, Karplus K, Li W, Lopez R, McWilliam H, Remmert M, Söding J, Thompson JD, Higgins DG. Fast, scalable generation of high-quality protein multiple sequence alignments using Clustal Omega. *Mol Syst Biol* 2011;7:539

3. Söding J. Protein homology detection by HMM-HMM comparison. *Bioinformatics* 2005; 21: 951–960.

4. Katoh K, Standley DM. MAFFT Multiple Sequence Alignment Software Version 7: Improvements in performance and usability. *Mol Biol Evol* 2013;30:772–780.

5. Capella-Gutierrez S, Silla-Martinez JM, Gabaldon T. trimAl: a tool for automated alignment trimming in large-scale phylogenetic analyses.*Bioinformatics* 2009;25:1972-1973.

6. Le SQ, Gascuel O. An improved general amino acid replacement matrix. *Mol Biol Evol* 2008;25: 1307-1320.

7. Le SQ, Gascuel O, Lartillot N. Empirical profile mixture models for phylogenetic reconstruction. *Bioinformatics* 2008;24:2317-2323.

8. Lartillot N, Philippe H. A Bayesian Mixture model for across-site heterogeneities in the amino-acid replacement process. *Mol Biol Evol* 2004;21:1095-1109.

9. Letunic I, Bork P. Interactive tree of life (iTOL) v3: an online tool for the display and annotation of phylogenetic and other trees. *Nucleic Acids Res* 44:W242-W245.

10. Jones P, Binns D, Chang H-Y, Fraser M, Li W, McAnulla C, McWilliam H, Maslen J, Mitchell A, Nuka G, Pesseat S, Quinn AF, Sangrador-Vegas A, Scheremetjew M, Yong S-Y, Lopez R, Hunter S. InterProScan 5: genome-scale protein function classification. *Bioinformatics* 2014;30:1236–1240.

11. Nguyen L-T, Schmidt HA, von Haeseler A, Minh BQ. IQ-TREE: A fast and effective stochastic algorithm for estimating maximum likelihood phylogenies.. *Mol Biol Evol* 2015; 32:268-274.

12. Wang HC, Minh BQ, Susko S, Roger AJ. Modeling site heterogeneity with posterior mean site frequency profiles accelerates accurate phylogenomic estimation. *Syst Biol* 2018;67:216–235.

13. Asgari S, Davis J, Wood D, Wilson P, McGrath A. Sequence and organization of the Heliothis virescens ascovirus genome. *J Gen Virol* 2007;88:1120-1132.

14. Bigot Y, Renault S, Nicolas J, Moundras C, Demattei MV, Samain S, Bideshi DK, Federici BA. Symbiotic virus at the evolutionary intersection of three types of large DNA viruses; iridoviruses, ascoviruses, and ichnoviruses. *PLoS ONE* 2009;4,e6397.

15.Wang L, Xue J, Seaborn CP, Arif BM, Cheng XW. Sequence and organization of the Trichoplusia ni ascovirus 2c (*Ascoviridae*) genome. *Virology* 2006;354:167-177.

16. Bigot Y, Renault S, Nicolas J, Moundras C, Demattei MV, Samain S, Bideshi DK, Federici BA. Symbiotic virus at the evolutionary intersection of three types of large DNA viruses; iridoviruses, ascoviruses, and ichnoviruses. *PLoS ONE* 2009;4:e6397.

17. Yanez RJ, Rodriguez JM, Nogal ML, Yuste L, Enriquez C, Rodriguez JF, Vinuela E. Analysis of the complete nucleotide sequence of African swine fever virus. *Virology* 1995;208:249-278.

18. Delhon G, Tulman ER, Afonso CL, Lu Z, Becnel JJ, Moser BA, Kutish GF, Rock DL. Genome of invertebrate iridescent virus type 3 (mosquito iridescent virus). *J Viro*l 2006;80:8439-8449.

19. Jakob NJ, Muller K, Bahr U, Darai G. Analysis of the first complete DNA sequence of an invertebrate iridovirus: coding strategy of the genome of Chilo iridescent virus. *Virology,* 2009;286:182-196.

20. Piegu B, Guizard S, Yeping T, Cruaud C, Asgari S, Bideshi DK, Federici BA, Bigot Y. Genome sequence of a crustacean iridovirus, IIV31, isolated from the pill bug, *Armadillidium vulgare. J Gen Virol* 2014;95:1585-1590.

21. Piegu B, Guizard S, Bideshi D, Spears T, Federici BA, Bigot Y. Unpublished.

22. Piegu B, Guizard S, Spears T, Cruaud C, Couloux A, Bideshi DK, Federici BA, Bigot Y. Complete genome sequence of invertebrate iridescent virus 22 isolated from a blackfly larva. *J Gen Virol* 2013;94:2112-2116.

23. Piegu B, Guizard S, Spears T, Cruaud C, Couloux A, Bideshi DK, Federici BA, Bigot Y. Complete genome sequence of invertebrate iridovirus IIV-25 isolated from a blackfly larva. *Arch Virol* 2013;159:1181-1185.

24. Wong CK, Young VL, Kleffmann T, Ward VK. Genomic and proteomic analysis of invertebrate iridovirus type 9. *J Virol* 2011;85:7900-7911.

25. Tong Y, Zhang J, Huang Y, Li S, Pei G, Zhang Z, Mi Z, An X. Unpublished.

26. Zhang QY, Xiao F, Xie J, Li ZQ, Gui JF. Complete genome sequence of lymphocystis disease virus isolated from China. *J Virol* 2004;78:6982-6994.

27. Tidona CA, Darai G. The complete DNA sequence of lymphocystis disease virus. *Virology* 1997;230:207-216.

28. He JG, Deng M, Weng SP, Li Z, Zhou SY, Long QX, Wang XZ, Chan SM. Complete genome analysis of the mandarin fish infectious spleen and kidney necrosis iridovirus. *Virology* 2001;291:126-239.

29. Jancovich JK, Mao J, Chinchar VG, Wyatt C, Case ST, Kumar S, Valente G, Subramanian S, Davidson EW, Collins JP, Jacobs BL. Genomic sequence of a ranavirus (family *Iridoviridae*) associated with salamander mortalities in North America. *Virology* 2003;316:90-103.

30. Jancovich JK, Bremont M, Touchman JW, Jacobs BL. Evidence for multiple recent host species shifts among the Ranaviruses (family *Iridoviridae*). *J Virol* 2010:84;2636-2647.

31. Mavian C, Lopez-Bueno,A, Fernandez Somalo,M.P, Alcami,A. And Alejo,A. Complete genome sequence of the European sheatfish virus. *J Virol* 2012;86:6365-6366.

32. Tan WG, Barkman TJ, Gregory Chinchar V, Essani,K. Comparative genomic analyses of frog virus 3, type species of the genus *Ranavirus* (family *Iridoviridae*). *Virology* 2003;323:70-84.

33. Song WJ, Qin QW, Qiu J, Huang CH, Wang F, Hew CL. Functional genomics analysis of Singapore grouper iridovirus: complete sequence determination and proteomic analysis. *J Virol* 2020;78:12576-12590.

34. Subramaniam K, Toffan A, Cappellozza E, Steckler NK, Olesen NJ, WaltzekTB. Unpublished.

35. de Groof A, Guelen L, Deijs M, van der Wal Y, Miyata M, Ng KS, van Grinsven L, Simmelink B, Biermann Y, Grisez L, van Lent J, de Ronde A, Chang SF, Schrier C, van der Hoek L. A Novel Virus Causes Scale Drop Disease in *Lates calcarifer. PLoS Pathog*. 2015; 11:e1005074.

36. Boyer M, Yutin N, Pagnier I, Barrassi L, Fournous G, Espinosa L, Robert C, Azza S, Sun S, Rossmann MG, Suzan-Monti M, La Scola B, Koonin EV, Raoult D. Giant Marseillevirus highlights the role of amoebae as a melting pot in emergence of chimeric microorganisms. *Proc Natl Acad Sci USA* 2009;106:21848-21853.

37. Thomas V, Bertelli C, Collyn F, Casson N, Telenti A, Goesmann A, Croxatto A, Greub G. Lausannevirus, a giant amoebal virus encoding histone doublets. *Environ Microbiol* 2011;13:1454-1466.

38. Doutre G, Philippe N, Abergel C, Claverie JM. Genome analysis of the first *Marseilleviridae* representative from Australia indicates that most of its genes contribute to virus fitness. *J Virol* 2014;88:14340-14349.

39. Takemura M. Draft genome sequence of tokyovirus, a member of the family *Marseilleviridae* isolated from the Arakawa river of Tokyo. *Japan Genome Announc* 2016;4:e00429-16.

40. Fischer MG, Allen MJ, Wilson WH, Suttle CA. Giant virus with a remarkable complement of genes infects marine zooplankton. *Proc Natl Acad Sci USA* 2010;107: 19508-19513.

41. Legendre M, Santini S, Rico A, Abergel C, Claverie JM. Breaking the 1000-gene barrier for mimivirus using ultra-deep genome and transcriptome sequencing. *Virol J* 2011;8:99.

42. Yoosuf N, Yutin N, Colson P, Shabalina SA, Pagnier I, Robert C, Azza S, Klose T, Wong J, Rossmann MG, La Scola B, Raoult D, Koonin EV. Related giant viruses in distant locations and different habitats: *Acanthamoeba polyphaga* moumouvirus represents a third Lineage of the *Mimiviridae* that Is close to the megavirus lineage. *Genome Biol Evol* 2012;4:1324-1330.

43. Arslan D, Legendre M, Seltzer V, Abergel C, Claverie JM. Distant mimivirus relative with a larger genome highlights the fundamental features of *Megaviridae*. *Proc Natl Acad Sci USA* 2011;108:17486-17491.

44. Legendre M, Lartigue A, Bertaux L, Jeudy S, Bartoli J, Lescot M, Alempic JM, Ramus C, Bruley C, Labadie K, Shmakova L, Rivkina E, Coute Y, Abergel C, Claverie JM. In-depth study of Mollivirus sibericum, a new 30,000-y-old giant virus infecting *Acanthamoeba*. *Proc Natl Acad Sci USA 2015;*112:E5327-E5335.

45. Philippe N, Legendre M, Doutre G, Coute Y, Poirot O, Lescot M, Arslan D, Seltzer V, Bertaux L, Bruley C, Garin J, Claverie JM, Abergel C. Pandoraviruses: amoeba viruses with genomes up to 2.5 Mb reaching that of parasitic eukaryotes. *Science*, 2013;341:281-286.

46. Scheid P. Viruses in close associations with free-living amoebae. *Parasitol Res* 2015;114:3959-3967.

47. Fitzgerald LA, Graves MV, Li X, Pfitzner AJP, Hartigan J, Van Etten JL. Sequence and annotation of the 288-kb ATCV-1 virus that infects an endosymbiotic *Chlorella* strain of the heliozoon *Acanthocystis turfacea. Virology* 2007;362:350-361.

48. Yanai-Balser GM, Duncan GA, Eudy JD, Wang D, Li X, Agarkova IV, Dunigan DD, Van Etten JL. Microarray analysis of *Paramecium bursaria* chlorella virus 1 transcription. *J Virol* 2010;84: 532-542.

49. Fitzgerald LA, Graves MV, Li X, Feldblyum T, Nierman WC, Van Etten JL. Sequence and annotation of the 369-kb NY-2A and the 345-kb AR158 viruses that infect *Chlorella* NC64. *Virology* 2007;358:472-484.

50. Fitzgerald LA, Graves MV, Li X, Feldblyum T, Hartigan J, Van Etten JL. Sequence and annotation of the 314-kb MT325 and the 321-kb FR483 viruses that infect *Chlorella* Pbi. *Virology* 2007;358;459-471.

51. Wilson WH, Schroeder DC, Allen MJ, Holden MT, Parkhill J, Barrell BG, Churcher C, Hamlin N, Mungall K, Norbertczak H, Quail MA, Price C, Rabbinowitsch E, Walker D, Craigon M. Roy D, Ghazal P. Complete genome sequence and lytic phase transcription profile of a Coccolithovirus. *Science* 2005;309:1090-1092.

52. Delaroque N, Bothe G, Pohl T, Knippers R, Mueller DG, Boland W. The complete nucleotide sequence of the *Ectocarpus siliculosus* virus genome. *Virology* 2011;287:112-132.

53. Schroeder DC, Park Y, Yoon HM, Lee YS, Kang SW, Meints RH, Ivey RG, Choi TJ. Genomic analysis of the smallest giant virus--*Feldmannia* sp. Virus 158. *Virology* 2009;384:223-232.

54. Derelle E, Ferraz C, Escande ML, Eychenie S, Cooke R, Piganeau G, Desdevises Y, Bellec L, Moreau H, Grimsley N. Life-cycle and genome of OtV5, a large DNA virus of the pelagic marine unicellular green alga *Ostreococcus tauri*. *PLoS ONE* 2008;3:e2250.

55. Moreau H, Piganeau G, Desdevises Y, Cooke R, Derelle E, Grimsley N. Marine prasinovirus genomes show low evolutionary divergence and acquisition of protein metabolism genes by horizontal gene transfer. *J Virol* 2010;84:12555-12563.

56. Derelle E, Monier A, Cooke R, Worden A.Z, Grimsley NH, Moreau H. Diversity of Viruses Infecting the Green Microalga *Ostreococcus lucimarinus. J Virol* 2015;89;5812-5821.

57. Weynberg KD, Allen MJ, Ashelford K, Scanlan DJ, Wilson WH. From small hosts come big viruses: the complete genome of a second *Ostreococcus tauri* virus, OtV-1. *Environ Microbiol* 2009;11:2821-2839.

58. Weynberg KD, Allen MJ, Gilg IC, Scanlan DJ, Wilson WH. Genome sequence of *Ostreococcus tauri* Virus OtV-2 throws light on the role of picoeukaryote niche separation in the ocean. J. Virol. 2011;85:4520-4529.

59. Zhang W, Zhou J, Liu T, Yu Y, Pan Y, Yan S, Wang Y. Four novel algal virus genomes discovered from Yellowstone Lake metagenomes. *Sci Rep* 2015;5:15131.

60. Santini S, Jeudy S, Bartoli J, Poirot O, Lescot M, Abergel C, Barbe V, Wommack KE, Noordeloos AA, Brussaard CP, Claverie JM. Genome of *Phaeocystis globosa* virus PgV-16T highlights the common ancestry of the largest known DNA viruses infecting eukaryotes. *Proc Natl Acad Sci USA* 2013;110:10800-10805.

61. Moniruzzaman M, LeCleir GR, Brown CM, Gobler CJ, Bidle KD, Wilson WH, Wilhelm SW. Genome of brown tide virus (AaV), the little giant of the *Megaviridae*, elucidates NCLDV genome expansion and host-virus coevolution. *Virology* 2014;466-467:60-70.

62. Gallot-Lavallee L, Pagarete A, Legendre M, Santini S, Sandaa RA, Himmelbauer H, Ogata H, Bratbak G, Claverie JM. The 474-kilobase-pair complete genome sequence of CeV-01B, a virus infecting *Haptolina* (Chrysochromulina) *ericina* (Prymnesiophyceae). *Genome Announc* 2015;3: e01413-5.

63. Legendre M, Bartoli J, Shmakova L, Jeudy S, Labadie K, Adrait A, Lescot M, Poirot O, Bertaux L, Bruley C, Coute Y, Rivkina E, Abergel C, Claverie JM.Thirty-thousand-year-old distant relative of

giant icosahedral DNA viruses with a pandoravirus morphology. *Proc Natl Acad Sci USA* 2014;111:4274-4279.

64. Mitsuhashi W, Miyamoto K, Wada S. The complete genome sequence of the alphaentomopoxvirus *Anomala cuprea* entomopoxvirus, including its terminal hairpin loop sequences, suggests a potentially unique mode of apoptosis inhibition and mode of DNA replication. *Virology* 2014;452-453:95-116.

65. Bawden AL, Glassberg KJ, Diggans J, Shaw R, Farmerie W, Moyer RW. Complete genomic sequence of the *Amsacta moorei* entomopoxvirus: analysis and comparison with other poxviruses. *Virology* 2000;274:120-139.

66. Theze J, Takatsuka J, Li Z, Gallais J, Doucet D, Arif B, Nakai M, Herniou EA. New insights into the evolution of Entomopoxvirinae from the complete genome sequences of four entomopoxviruses infecting *Adoxophyes honmai*, *Choristoneura biennis*, *Choristoneura rosaceana*, and *Mythimna separata*. *J Virol* 2013;87:7992-8003.

67. Afonso CL, Tulman ER, Lu Z, Oma E, Kutish GF, Rock DL. The genome of *Melanoplus sanguinipes* entomopoxvirus. *J Virol* 1999;73:533-552.

68. Gjessing MC, Yutin N, Tengs T, Senkevich T, Koonin EV, Ronning HP, Alarson M, Ylving S, Lie K-I, Saure B, Tran L, Moss B, Dale,O.B. Salmon gill poxvirus, the deepest representative of the *Chordopoxvirinae*. *J Virol* 2015;89:9348-9367.

69. Tulman ER, Afonso CL, Lu Z, Zsak L, Kutish GF, Rock DL. The genome of canarypox virus. *J Virol* 2004;78:353-366.

70. Offerman K, Carulei O, van der Walt AP, Douglass N, Williamson AL. The complete genome sequences of poxviruses isolated from a penguin and a pigeon in South Africa and comparison to other sequenced avipoxviruses. BMC Genomics. 2014;15:463.

71. Banyai K, Palya V, Denes B, Glavits R, Ivanics E, Horvath B, Farkas SL, Marton S, Balint A, Gyuranecz M, Erdelyi K, Dan A. Unique genomic organization of a novel Avipoxvirus detected in turkey (*Meleagris gallopavo)*. I*nfect Genet Evol* 2015;35:221-229.

72. Afonso CL, Tulman ER, Lu Z, Zsak L, Kutish GF, Rock DL. The genome of fowlpox virus. *J Virol* 2000;74:3815-3831.

73. Tulman ER, Afonso CL, Lu Z, Zsak L, Sur JH, Sandybaev NT, Kerembekova UZ, Zaitsev VL, Kutish GF, Rock DL. The genomes of sheeppox and goatpox viruses. *J Virol* 2002;76:6054-6061.

74. Tulman ER, Afonso CL, Lu Z, Zsak L, Kutish GF, Rock DL. Genome of lumpy skin disease virus. *J Virol* 2001;75:7122-7130.

75. Zhao G, Droit L, Tesh RB, Popov VL, Little NS, Upton C, Virgin HW, Wang D. The Genome of Yoka poxvirus. 2011;85:10230-10238.

76 Afonso CL, Delhon G, Tulman ER, Lu Z, Zsak A, Becerra VM, Zsak L, Kutish GF, Rock DL. Genome of deerpox virus. *J Virol* 2005;79:966-977.

77. Afonso CL, Tulman ER, Delhon G, Lu Z, Viljoen GJ, Wallace DB, Kutish GF, Rock DL. Genome of crocodilepox virus. *J Virol* 2006;80:4978-4991.

78. Johnston JB, Barrett JW, Nazarian SH, Goodwin M, Ricciuto D, Wang G, McFadden G. A poxvirus-encoded pyrin domain protein interacts with ASC-1 to inhibit host inflammatory and apoptotic responses to infection. *Immunity* 2005;23:587-598.

79. Willer DO, McFadden G, Evans DH. The complete genome sequence of shope (rabbit) fibroma virus. *Virology* 1999;264:319-343.

80. Senkevich TG, Bugert JJ, Sisler JR, Koonin EV, Darai G, Moss B. Genome sequence of a human tumorigenic poxvirus: prediction of specific host response-evasion genes. *Science* 1996;273:813-816.

81. Afonso CL, Tulman ER, Lu Z, Zsak L, Zaitsev VL, Kerembekova UZ, Sandybaev NT, Kutish GF, Rock DL. The genome of camelpox virus. *Virology* 2002;295:1-9.

82. Hu FQ, Smith CA, Pickup DJ. Cowpox virus contains two copies of an early gene encoding a soluble secreted form of the type II TNF receptor. *Virology* 1994;204:343-356.

83. Chen N, Buller RM, Wall EM, Upton C. Analysis of host response modifier ORFs of ectromelia virus, the causative agent of mousepox. *Virus Res* 2000;66:155-173.

84. Shchelkunov SN, Totmenin AV, Babkin IV, Safronov PF, Ryazankina OI, Petrov NA, Gutorov VV, Uvarova EA, Mikheev MV, Sisler JR, Esposito JJ, Jahrling PB, Moss B, Sandakhchiev LS. Human monkeypox and smallpox viruses: genomic comparison. *FEBS Lett* 2001;509:66-70.

85. Fleischauer C, Upton C, Victoria J, Jones GJ, Roper RL. Genome sequence and comparative virulence of raccoonpox virus: The first North American poxvirus sequence. *J Gen Virol* 2015;96:2806-28021.

86. Esposito JJ, Sammons SA, Frace AM, Osborne JD, Olsen-Rasmussen M, Zhang M, Govil D, Damon IK, Kline R, Laker M, Li Y, Smith GL, Meyer H, Leduc JW, Wohlhueter RM. Genome sequence diversity and clues to the evolution of variola (smallpox) virus. Science 2006;313:807-812.

87. Esposito JJ, Moss B, Sammons SA, Frace AM, Olsen-Rasmussen M, Osborne J, Baroudy BM, Wohlhueter R. Direct Submission.

88. Shchelkunov SN, Totmenin AV, Sandakhchiev LS. Analysis of the nucleotide sequence of 23.8 kbp from the left terminus of the genome of variola major virus strain India-1967. *Virus Res* 1996;40:169-183.

89. Delhon G, Tulman ER, Afonso CL, Lu Z, de la Concha-Bermejillo A, Lehmkuhl HD, Piccone ME, Kutish GF, Rock DL. Genomes of the parapoxviruses ORF virus and bovine papular stomatitis virus. *J Virol* 2004;78:168-177.

90. Friederichs S, Stefan K, Helmut B, Heike L, Mathias B. Parapoxvirus (PPV) of red deer reveals sub-clinical infection and confirms a unique species. *J Gen Virol* 2015;96:1446-1462.

91. Hautaniemi M, Ueda N, Tuimala J, Mercer AA, Lahdenpera J, McInnes,C.J. The genome of pseudocowpoxvirus: comparison of a reindeer isolate and a reference strain. *J Gen Virol* 2010;91:1560-1576.

92. Afonso CL, Tulman ER, Lu Z, Zsak L, Osorio FA, Balinsky C, Kutish GF, Rock DL. The genome of swinepox virus. *J Virol* 2002;76:783-790.

93. Brunetti CR, Amano H, Ueda Y, Qin J, Miyamura T, Suzuki T, Li X, Barrett JW, McFadden G. Complete genomic sequence and comparative analysis of the tumorigenic poxvirus yaba monkey tumor virus. *J Virol* 2003;77:13335-13347.

94. Lee HJ, Essani K, Smith GL. The genome sequence of Yaba-like disease virus, a yatapoxvirus. *Virology* 2001;281:170-192.

95. Darby AC, McInnes CJ, Kjaer KH, Wood AR, Hughes M, Martensen PM, Radford AD, Hall N, Chantrey J.

96. Afonso PP, Silva PM, Schnellrath LC, Jesus DM, Hu J, Yang Y, Renne R, Attias M, Condit RC, Moussatche N, Damaso CR. Biological characterization and next-generation genome sequencing of the unclassified cotia virus SPAn232 (*Poxviridae*). *J Virol*, 2012;86:5039-5054.

97. Rost B. Twilight zone of protein sequence alignments. *Protein Eng Des Sel* 1999;12:85-94.

98. Yutin N, Wolf YI, Raoult D, Koonin EV. Eukaryotic large nucleo-cytoplasmic DNA viruses: clusters of orthologous genes and reconstruction of viral genome evolution. *Virol J*. 2009; 6:223.

99. Townsley AC, Senkevich TG, Moss B. Vaccinia virus A21 virion membrane protein is required for cell entry and fusion. *J Virol* 2005; 79: 9458-9469.

100. Osborne RJ, Symonds TM, Sriskantha A, Lai-Fook J, Fernon CA, Dall DJ. An entemopoxvirus homologue of the vaccinia virus D13L-encoded ‘rifampicin resistance’ protein. *J Gen Virol* 1996; 77: 839-346.

101. Senkevich TG, Ward BM, Moss B. Vaccinia virus A28L gene encodes an essential protein component of the virion membrane with intramolecular disulfide bonds formed by the viral cytoplasmic redox pathway. *J Virol* 2004; 78: 2348-2356.

102. Finn RD, Coggill P, Eberhardt RY, Eddy SR, Mistry J, Mitchell AL, Potter SC, Punta M, Qureshi M, Sangrador-Vegas A, Salazar GA, Tate J, Bateman A. The Pfam protein families database: towards a more sustainable future. *Nucleic Acids Res* 2016;44:D279-D285
